# Coupling of Spectrin Repeat Modules for the Assembly of Nanorods and Presentation of Protein Domains

**DOI:** 10.1021/acsnano.4c07701

**Published:** 2024-10-11

**Authors:** Klemen Mezgec, Jaka Snoj, Liza Ulčakar, Ajasja Ljubetič, Magda Tušek Žnidarič, Miha Škarabot, Roman Jerala

**Affiliations:** † Department of Synthetic Biology and Immunology, National Institute of Chemistry, SI-1000 Ljubljana, Slovenia; ‡ Graduate School of Biomedicine, University of Ljubljana, SI-1000 Ljubljana, Slovenia; § EN-FIST Centre of Excellence, SI-1000 Ljubljana, Slovenia; ∥ Department of Biotechnology and Systems Biology, National Institute of Biology, SI-1000 Ljubljana, Slovenia; ⊥ Condensed Matter Department, Jozef Stefan Institute, SI-1000 Ljubljana, Slovenia; # CTGCT, Centre of Technology of Gene and Cell Therapy, Hajdrihova 19, SI-1000 Ljubljana, Slovenia

**Keywords:** polypeptide nanostructures, spectrin repeat, dystrophin, protein self-assembly, protein rods, biomaterials, adjustable spacing

## Abstract

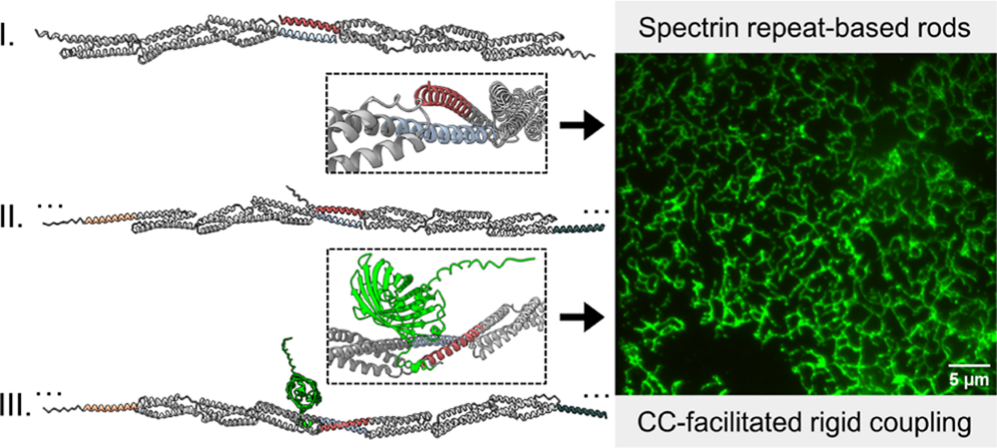

Modular protein engineering is a powerful approach for fabricating high-molecular-weight assemblies and biomaterials with nanoscale precision. Herein, we address the challenge of designing an extended nanoscale filamentous architecture inspired by the central rod domain of human dystrophin, which protects sarcolemma during muscle contraction and consists of spectrin repeats composed of three-helical bundles. A module of three tandem spectrin repeats was used as a rigid building block self-assembling via coiled-coil (CC) dimer-forming peptides. CC peptides were precisely integrated to maintain the spectrin α-helix continuity in an appropriate frame to form extended nanorods. An orthogonal set of customizable CC heterodimers was harnessed for modular rigid domain association, which could be additionally regulated by metal ions and chelators. We achieved a robust assembly of rigid rods several micrometers in length, determined by atomic force microscopy and negative stain transmission electron microscopy. Furthermore, these rigid rods can serve as a scaffold for the decoration of diverse proteins or biologically active peptides along their length with adjustable spacing up to tens of nanometers, as confirmed by the DNA-PAINT super-resolution microscopy. This demonstrates the potential of modular bottom-up protein engineering and tunable CCs for the fabrication of functionalized protein biomaterials.

## Introduction

Proteins serve as fundamental building blocks and molecular machines of all cells, with their complexity emerging from the directed self-assembly of monomers into highly organized supramolecular structures.^1,2^ Many protein-based materials exhibit attractive functional properties.^3^ Proteins can engage in an extensive network of interactions among themselves and with other molecules. This makes proteins nature’s most diverse and versatile programmable biopolymers, consisting of linear amino acid chains capable of spontaneous folding into defined three-dimensional (3D) structures.^4,5^ The interactions within individual or multiple molecules enable proteins to function either as a standalone unit or as an integral component of complex assemblies.^5,6^ The diversity of protein assemblies that evolved through natural selection serves protein engineers as a source of inspiration to construct designed proteins.^7−9^ The bottom-up design of nanostructures offers a strategy for constructing tailor-made high-molecular-weight assemblies with predefined spatial configurations.^10,11^ Hitherto, strategies of rational protein engineering have been successfully implemented in the construction of artificial supramolecular protein assemblies of various geometries, ranging from one-dimensional (1D) filaments,^12−20^ two-dimensional (2D) tubes,^21−23^ and lattices,^24−26^ to more complex 3D structures, such as hydrogels,^27^ protein cages,^28,29^ and multidomain virus-like particles.^30,31^

Filamentous biopolymers represent an attractive material for bionanotechnology.^21,32,33^ Nature offers several proteins that fold into filamentous structures, primarily to provide structural integrity and mechanical resilience to specialized cells, as well as to facilitate the functions of cellular machinery.^33,34^ Such proteins are often very large, with repetitive sequences, and assembled in a coordinated and complex multistep process.^33,35^ As a result, reverse engineering and production of high-molecular-weight artificial protein assemblies with programmable functionalities remains challenging.^8^ To circumvent these challenges, we were inspired by the family of filamentous scaffolding proteins comprising spectrin repeats, which are components of the membrane cytoskeleton and actin bundling,^36,37^ including spectrin, dystrophin, α-actinin, and many others. Dystrophin is a large protein (429 kDa), expressed in cardiac and skeletal muscle cells that interacts with several cytosolic as well as plasma membrane proteins.^38−40^ It is responsible for establishing and maintaining a structural connection between the extracellular matrix and skeletal actin.^38,40^ Moreover, dystrophin also plays a role in the resistance to mechanical shear forces in myocytes.^40,41^ It is composed of four major domains, the N-terminal actin-binding domain (ABD1), elongated central rod-like domain, cysteine-rich domain (CRD), and C-terminal binding domain (CTD).^40^ The central rod domain comprises 24 spectrin modules.^41,42^ Each spectrin module is made of ∼105 amino acid residues, forming three helices, where the third helix is continuous with the first helix of the next repeat, an arrangement that imparts rigidity to the filament.^41,42^ This geometry provides interesting mechanical properties and involvement in maintaining muscle tissue integrity. The tandemly repeated spectrin module, therefore, represents a natural archetype for modular protein assemblies that could be translated to various biomedical and nanostructural settings.

We reasoned that coiled-coil (CC) dimers could be used as connectors that can rigidly couple spectrin domains. CC dimers offer a versatile and reliable alternative to complex and often case-specific protein–protein interfaces.^43^ They are well-understood structural motifs, characterized by a heptad repeat pattern.^43−46^ They are composed of two or more α-helices, twisted into a left-handed superhelix, driven by a combination of hydrophobic (residues at positions *a* and *d*) and electrostatic (positions *e* and *g*) interactions.^47,48^ Other positions of the heptad repeat (*b*, *c*, and *f*) are usually occupied by residues with high helical propensity that may form intramolecular salt bridges, contributing to thermodynamic stability.^44,47,49^ Due to their pairing specificity, predictability, and customizability, CCs have been utilized as designable building blocks^28,29,50−53^ or linking motifs^54,55^ for engineered modular assemblies in various structural geometries and sizes. Moreover, CCs can be engineered to possess a metal-ion coordination site, where the positioning of His residues with simultaneous destabilization of the hydrophobic core introduces a conformational switch, which can induce the formation of CC dimers depending on the presence of Zn(II) ions (switCCh).^56^ The metal-ion-responsive CCs were used as metal-regulated building blocks for the self-assembly of CC protein origami (CCPO) triangles or bipyramidal protein cages.^57^ The versatility, structural simplicity, well-defined interaction surfaces, discrete rules of their oligomerization nature, and stability of CC dimers led to a decision to incorporate them for fibrillar protein assembly.^43,44,49^ Nanoassemblies with decoration features separated by tens of nanometers could be useful for modulating biological activity, such as, e.g., activating B-cell receptors, as demonstrated in vaccines based on virus-like particles or DNA nanostructures with optimal separations around 20 nm.^58−60^ Therefore, engineered self-assembling filaments with adjustable spacing in the order of tens of nanometers could be useful in biotechnology, bioengineering, and biomedicine.

Here, we combined a module composed of three spectrin repeats with orthogonal mono- and bifunctional CC-interacting domains for rigid coupling, thereby maintaining the continuity of spectrin helices for the formation of extended rods. Based on the favorable folding, stability, and pairwise affinity of CC dimer modules, we used them for the construction of supramolecular assemblies with a filamentous architecture. The designs were supported by Alphafold2 (AF2)^61^ molecular models with high confidence metrics. The molecular models emphasized the key role of the linker design in preserving structural rigidity by maintaining α-helix continuity from the terminal helix of the scaffolding spectrin domain across the CC-forming peptide within the individual building block in an appropriate frame to ensure rigid extension. We demonstrate that engineered assemblies can serve as scaffolds for the spatial arrangement and presentation of protein domains on filaments with adjustable spacing. Through the implementation of Zn(II)-responsive CC-dimerizing segments, we demonstrate reversible control of the assembly and disassembly of engineered structures.

## Results

### Protein Design of Self-Assembling Spectrin Repeat-Based Building Blocks

**Figure 1:**
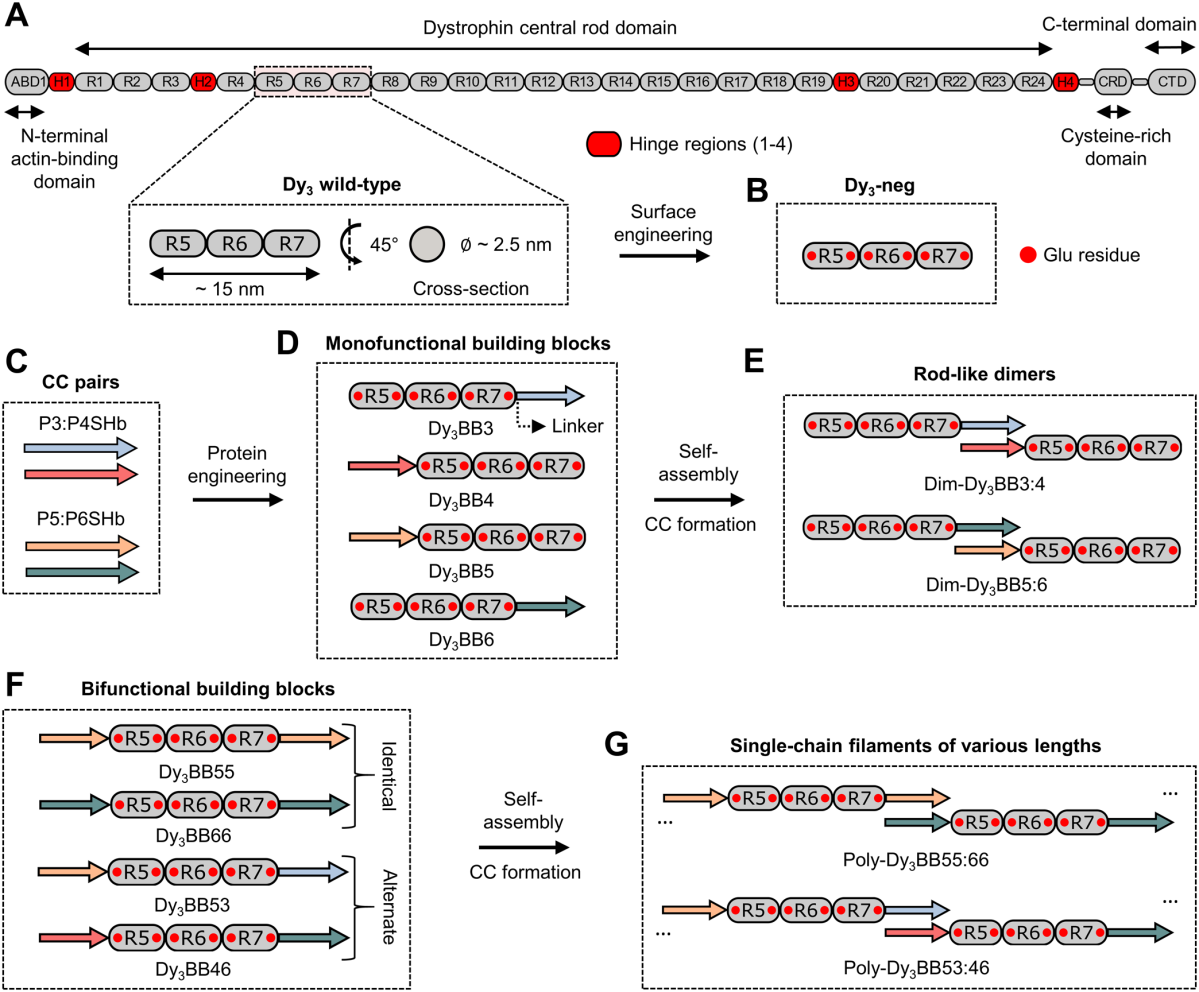
Schematic representation of the design and the strategy of CC-facilitated self-assembly of spectrin repeat-based filaments. (A) Schematic representation of the human cytoskeletal protein dystrophin. Its structure is composed of four main domains: N-terminal ABD1, central rod domain, CRD, and CTD. Hinge regions are colored red (H1–4). A wild-type array of three tandem spectrin repeats is magnified and denoted as Dy_3_ wild-type (R5-R7). (B) Surface engineering of the scaffold was performed by mutating six exposed Gly to Glu residues. The resulting protein domain was named Dy_3_-neg. Mutated Glu residues are schematically depicted with red dots. (C) CC-forming peptides are presented in specific colors (P3SHb in light blue, P4SHb in red, P5SHb in orange, and P6SHb in green). (D) Protein engineering of monofunctional building blocks designed to self-assemble into dimers. The position of the representative ultrashort helical linker is depicted with a black dotted arrow. All building blocks utilized in this study follow the universal naming convention Dy_r_BBXX. Dy stands for dystrophin, _r_ is the number of repeats in the central scaffolding unit, BB is the abbreviation for the building block, and XX denotes the type of fused CC-forming peptide. (E) Schematic representation of dimers, self-assembled from monofunctional blocks Dy_3_BB3, Dy_3_BB4, Dy_3_BB5, and Dy_3_BB6. Dimer assemblies are named Dim-Dy_3_BBX:X. Folded CC motifs are denoted with the colon between peptide numbers (e.g., Dim-Dy_3_BB3:4). (F) Design of bifunctional building blocks utilized in this study. CC-forming peptides are fused at both terminal ends of the Dy_3_-neg complex in two different structural arrangements (identical or alternate). (G) Schematic presentation of polymerization into single-chain filaments. Filamentous assemblies follow a similar naming convention to other designs. An example is Poly-Dy_r_BBXX:XX, where Poly denotes the polymer type and XX:XX represents the specific CC pairing between peptides (e.g., 55:66).

The central self-assembling scaffolding unit for extendable filamentous biopolymers consisted of three tandem spectrin repeats (Dy_3_), derived from the elongated central rod domain of human dystrophin, which we deemed suitable for the construction of fibrillar assemblies to mimic the natural filamentous geometry (Figure 1A). The high-resolution structure of the full-length dystrophin central rod domain remains unresolved. However, small-angle X-ray scattering (SAXS) analysis in solution indicated an extended shape of several spectrin repeats, similar to other high-resolution elongated structures comprising 2–4 spectrin modules.^42^ AF2 was used to generate a molecular model of the wild-type Dy_3_ module (Figure S1A), which revealed a rigid linear array of three tandem repeats of the triple α-helical bundle, each measuring ∼5 nm in length. The Dy_3_ measures ∼15 nm in length and ∼2.5 nm in diameter (Figures 1A and S1A,B), making it suitable for the construction of assemblies with a filamentous architecture.

To mitigate the challenges of producing soluble protein, 6 solvent-exposed residues were replaced with Glu residues, which have high helical propensity and are charged, to engineer a supercharged variant named Dy_3_-neg. Substitution decreased the net charge at neutral pH from −4.8 for Dy_3_ to −10.8 for Dy_3_-neg (Figures 1B and S1B,C). The Dy_3_-neg was produced in *Escherichia coli* as a soluble protein and purified to high yield (∼40 mg/L) by Ni^2+^-affinity (Ni-NTA) and size exclusion chromatography (SEC). Sodium dodecyl sulfate-polyacrylamide gel electrophoresis (SDS-PAGE) confirmed the correct size and purity of the isolated protein (Figure S2A). The purified Dy_3_-neg was monodisperse in solution with a molecular weight determined by size exclusion chromatography coupled to multiangle light scattering (SEC-MALS) of 40 kDa, in close agreement with the theoretical value (39.6 kDa) (Figure S2B and Table S3). The circular dichroism (CD) spectrum corresponded to a typical α helical secondary structure with a melting temperature (*T*m) of 55 °C. The protein refolded efficiently by cooling from 90 to 25 °C (Figure S2C,D). Additionally, dynamic light scattering (DLS) confirmed the monodispersity in the buffer solution (Figure S2E). The SAXS profile matched well the molecular model (χ2 = 1.85) (Figure S3A,C), and the ab initio shape model calculated from the scattering curve agreed with the molecular model (Figure S3C). The distance distribution function exhibited a tail at large *r* characteristic for elongated particles,^42^ with the determined *D*_max_ of 22.4 nm (Figure S3B). These results indicated that Dy_3_-neg was indeed rigidly elongated, making it suitable for use as a scaffolding domain to guide the assembly of filamentous architectures.

To introduce the directed oligomerization capacity to Dy_3_-neg, we implemented complementary parallel heterodimeric CC-forming peptide pairs at each end to connect spectrin repeat-based modules (Figure 1C,D). We reasoned that the CC motif could serve as a modular, specific, stable, and tunable linking segment between adjacent Dy_3_-neg modules. CC-forming peptides were fused to terminal helices of Dy_3_-neg to generate reactive mono- (one peptide) or bifunctional (two peptides) protein blocks (Figure 1D,F). The coupling of CC-dimer forming peptides to the spectrin domain was investigated for linkers spanning from 0 to 6 residues, aiming to optimize the relative orientation of the neighboring spectrin repeat modules to form an extended rigid chain. Based on AF2 3D models, we selected a linker comprising a single Glu residue, with high helical propensity as an ultrashort linker to preserve the continuity of terminal α-helices of trimeric spectrin repeat-based subunits (Figures 1D and S4A,B). This was expected to result in the rigidity of the joining regions and enable sufficient spacing to form fully folded CC-linking motifs between the neighboring units (Figure S4A,B). Mono- and bifunctional building blocks were designed for dimerization and intended polymerization into filamentous assemblies, mimicking the structural arrangement of the dystrophin central rod domain (Figure 1E,G).

### Design Principles and Biophysical Characterization of CC Dimerization Motifs

**Figure 2:**
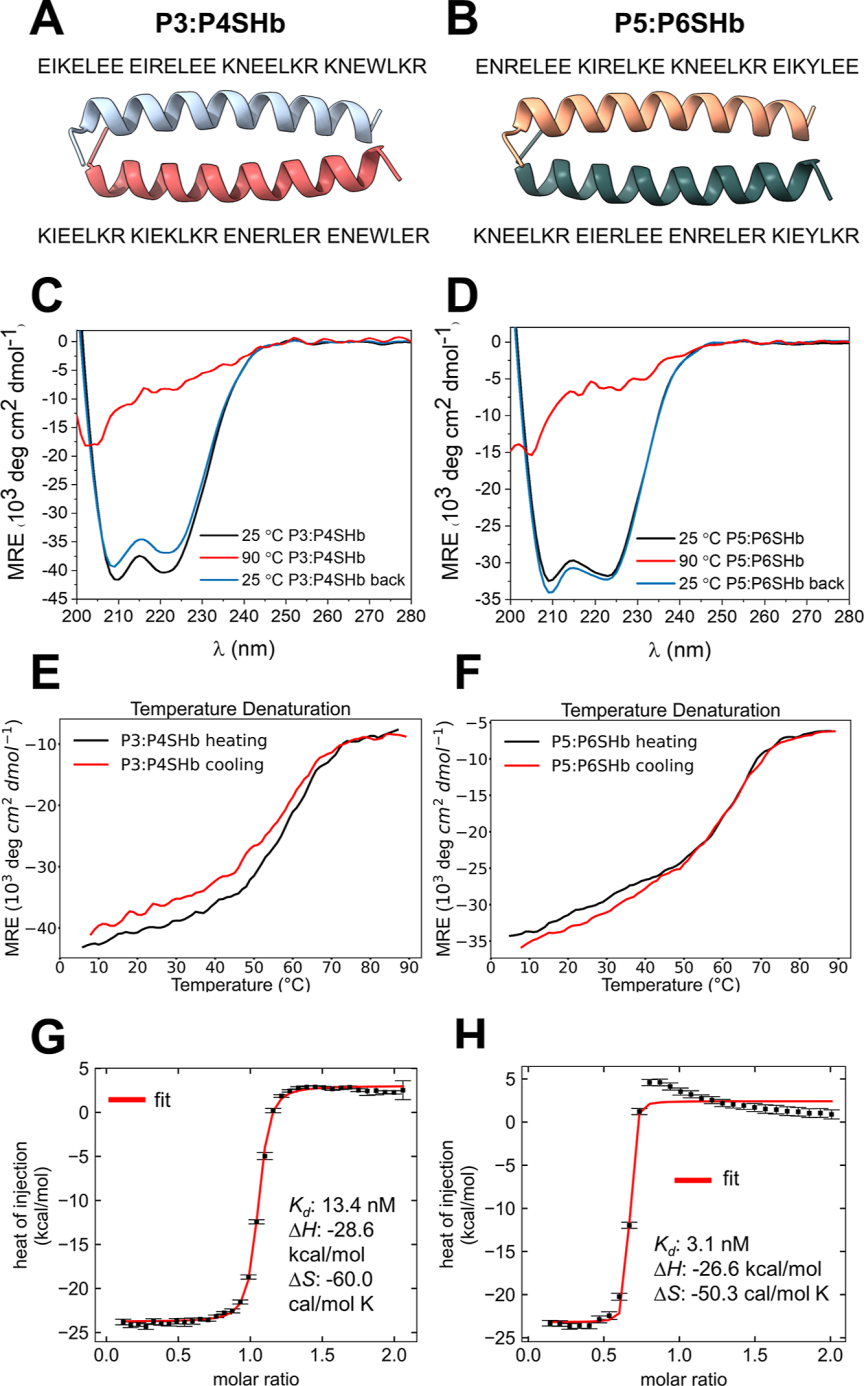
Design principles and biophysical characterization of designed CC heterodimeric pairs. (A, B) Amino acid sequence and models of designed CC pairs P3:P4SHb (A) and P5:P6SHb (B). Molecular models were made with AF2. (C–D) CD spectra of designed peptide pairs (20 μM). Measurements were performed at three different temperatures to probe the refolding ability of the CC motifs. Measurement at 25 °C is depicted with a black line and at 90 °C with a red line; the sample is cooled back to 25 °C with a blue line. The CD signal is shown as mean residue ellipticity. (E, F) Thermal melting was monitored with CD at 222 nm as a function of temperature. The black curve represents the heating of the sample, and the red curve represents cooling reversibility. Melting temperatures (*T*m) were determined by fitting the experimental measurements (black curve) to a thermodynamic sigmoidal curve. (G, H) Binding analysis of peptide partners using ITC measurements. ITC curves (red) were determined by fitting the experimental data points (black dots) to a 1:1 binding model.

The P3:4SHb and P5:6SHb heterodimeric parallel CC pairs (Figure 2A,B) were developed for higher stability and helical propensity than their predecessors.^49^ Their orthogonality was defined by matching Asn residues at the *a* position and oppositely charged Glu and Lys at the *e* and *g* positions (Figure S5A,B). We further increased their helicity and stability by introducing charged residues (Glu, Lys, and Arg) at the *b*, *c*, and *f* positions to introduce intramolecular salt bridges (Figures 2A,B and S5A,B). The peptide pairs were properly folded, exhibiting CD spectral characteristic of an α-helix (Figure 2C,D), and were able to refold when cooled after thermal denaturation (Figure 2C,D). Both designed CCs exhibited high thermal stability, with *T*m values of 60.4 °C for P3:P4SHb and 61.5 °C for P5:P6SHb (Figure 2E,F). Modeling of the complex provided structural insights (Figures 2A,B and S5A,B), and the affinity determined by isothermal titration calorimetry (ITC) was in the nM range (13.5 nM and 3.1 nM for P3:P4SHb and P5:P6SHb, respectively) (Figure 2G,H).

### Design and Biophysical Characterization of CC-Facilitated Dimer Assembly

**Figure 3:**
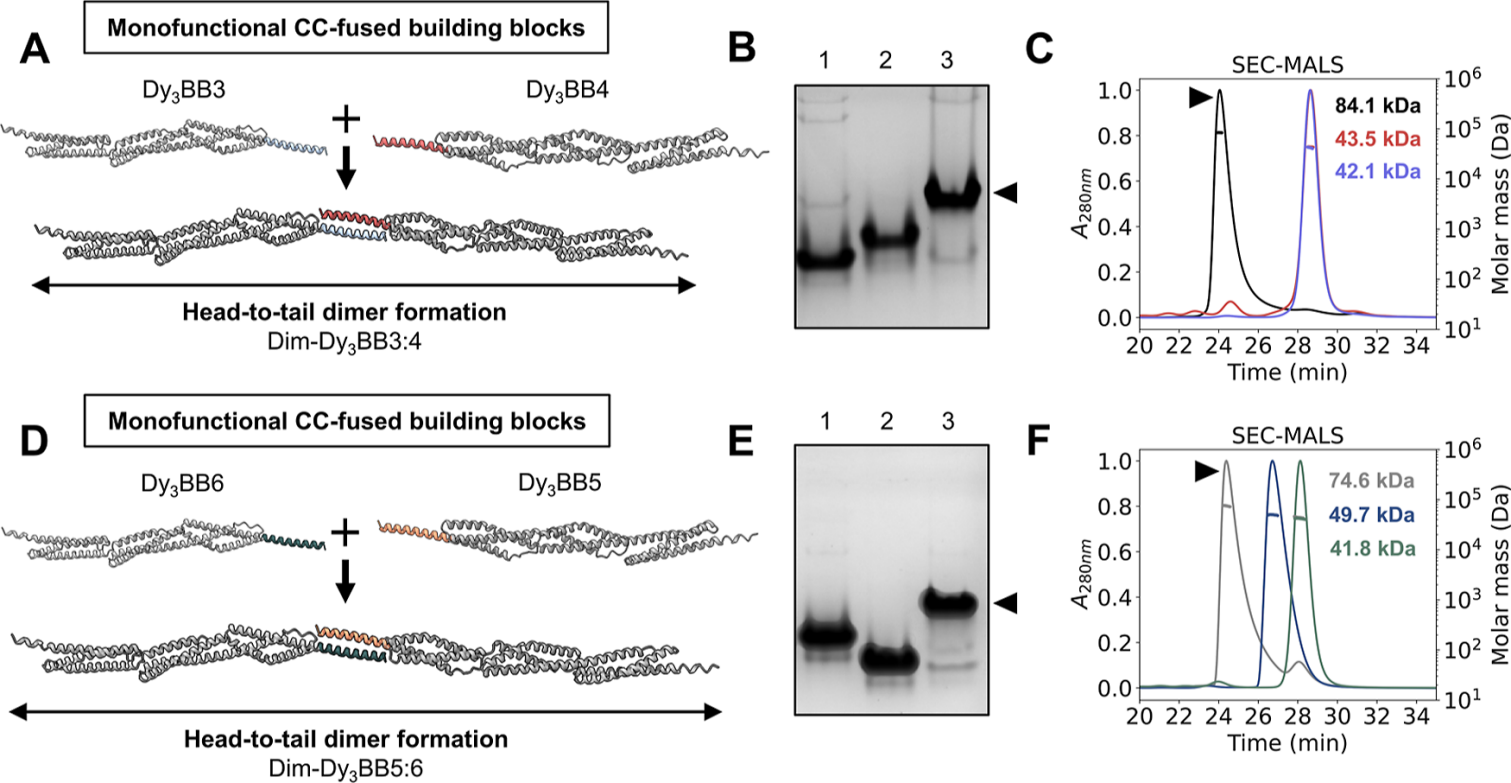
CC-facilitated self-assembly into extended dimers. (A,D) AF2 molecular models of the interacting monofunctional building blocks Dy_3_BB3 and Dy_3_BB4 or Dy_3_BB6 and Dy_3_BB5 that form dimers Dim-Dy_3_BB3:4 and Dy_3_BB5:6, respectively. (B) Native PAGE confirmed the dimerization of Dy_3_BB3 and Dy_3_BB4 into Dim-Dy_3_BB3:4 at 1:1 stoichiometry (each protein at 20 μM) (lane 3, black arrow). Lanes 1 and 2 represent the mobility of monomeric controls Dy_3_BB3 and Dy_3_BB4, respectively. (C,F) SEC-MALS chromatograms with indicated molecular weights of dimer assemblies Dim-Dy_3_BB3:4 (black line, black arrow) and Dim-Dy_3_BB5:6 (gray line, black arrow), as well as monomers Dy_3_BB3 (red line), Dy_3_BB4 (violet line), Dy_3_BB5 (blue line), and Dy_3_BB6 (green line), calculated from the light scattering data. (E) Native PAGE showcased dimerization of the equimolar mix of Dy_3_BB5 and Dy_3_BB6 (each protein at 20 μM) into Dim-Dy_3_BB5:6 (lane 3, black arrow). Lanes 1 and 2 display the mobility of individual monomers Dy_3_BB5 and Dy_3_BB6, respectively.

To thoroughly evaluate whether such an arrangement can result in an efficient head-to-tail self-assembly into dimers in an extended conformation, we tested two dimerization systems composed of four monofunctional building blocks comprising a scaffolding domain Dy_3_-neg fused to different CC-forming peptides (Figure 3A,D). To ensure the appropriate geometry without steric interference and maintain seamless α-helix continuity from the spectrin domain, P3SHb or P6SHb was fused to the C-terminus (Dy_3_BB3 and Dy_3_BB6) and P4SHb or P5SHb to the N-terminus (Dy_3_BB4 and Dy_3_BB5) of the Dy_3_-neg. All isolated recombinant proteins exhibited >95% purity based on SDS-PAGE (Figures S6A and S7A) and a high α-helical secondary structure content (Figures S6B,D and S7B,D). Dy_3_BB3 and Dy_3_BB6 exhibited similar two-state unfolding, whereas Dy_3_BB4 and Dy_3_BB5 indicated a more complex transition (Figures S6C,E and S7C,E), demonstrating that the position and type of the peptide may influence the properties of individual building blocks.

To analyze the assembly into dimers (Figure S8A), we employed native PAGE, DLS, and SEC-MALS. Native PAGE showed that each building block changed from the monomeric state to a distinct band with slower mobility upon mixing (Dim-Dy_3_BB3:4 and Dim-Dy_3_BB5:6), indicating dimerization (Figure 3B,E). This feature was confirmed by SEC-MALS, with the measured molecular weight closely matching the calculated values (Figure 3C,F and Table S3). DLS analysis revealed that the hydrodynamic radius increased from ∼8–13 nm for monomers to ∼14 and 18 nm for Dim-Dy_3_BB3:4 and Dim-Dy_3_BB5:6, respectively (Figure S8B,C). Atomic force microscopy (AFM) imaging revealed structures with expected lengths (∼35 nm) for both dimers. However, height profiles are likely underestimated (measured ∼0.5 to 0.8 nm, versus theoretical ∼2.5 nm), most probably due to drying-induced sample deformation.^62^

Particles visualized by AFM exhibited an extended rod-like conformation, with some degree of bending, indicating that head-to-tail dimerization of engineered spectrin repeat-based trimer subunits can yield rod-like assemblies (Figure S8D,E).

### Construction of Artificial Microscale Rods by Harnessing Engineered Spectrin Repeat-Based Protein Blocks

**Figure 4:**
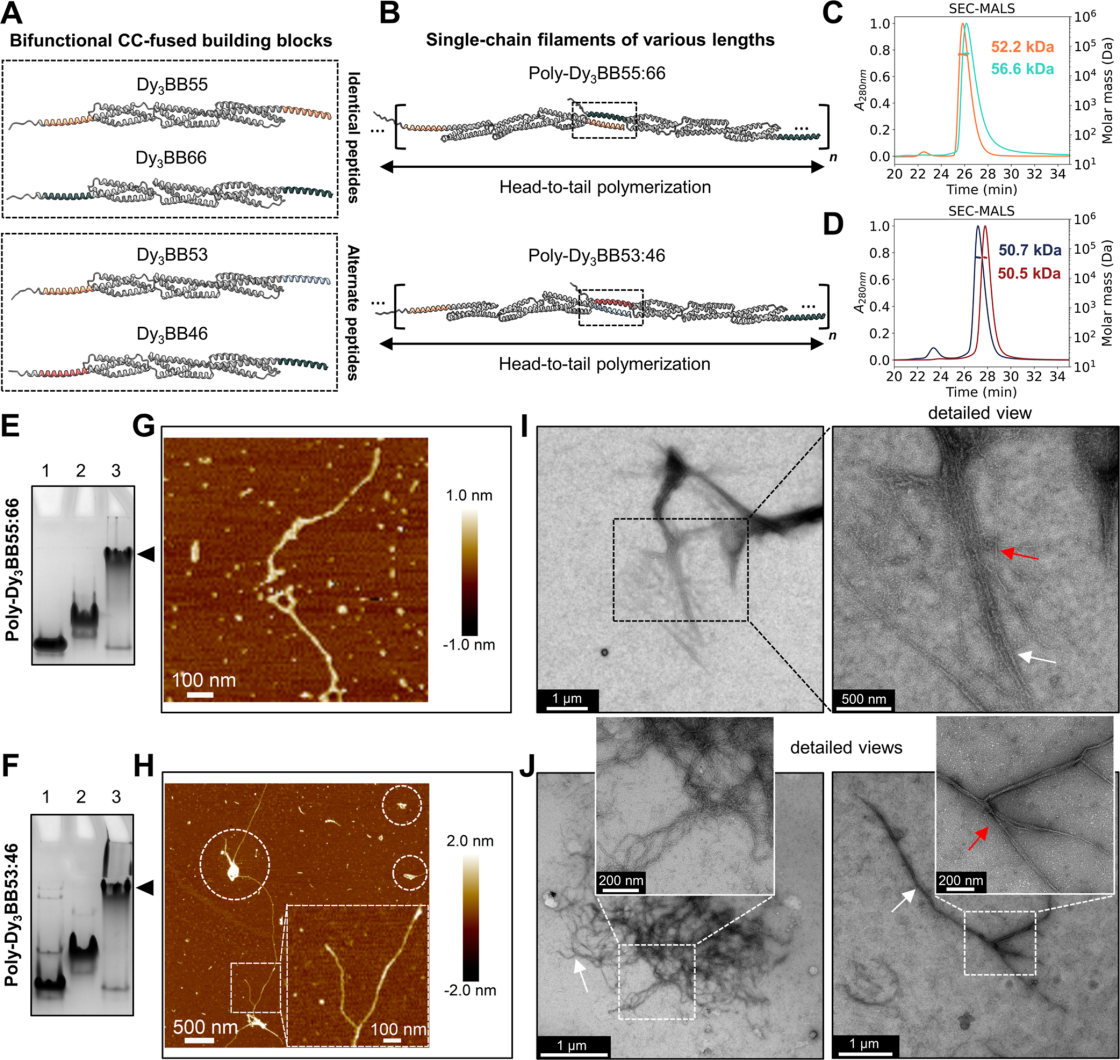
Bifunctional protein block design and construction of spectrin repeat-based rods. (A) Graphical representation of engineered bifunctional building blocks with identical or alternate peptide arrangement. (B) Proposed mechanism of extended head-to-tail polymerization into single-chain filaments facilitated by orthogonal CC-linking motifs. Protein folding, spatial positioning, and CC formation (black dashed rectangles) were validated by AF2 modeling. This structural arrangement is expected to be repeated multiple times in the final assembly (noted with *n*). (C,D) SEC-MALS chromatograms with indicated molecular weights of individual protein blocks Dy_3_BB55 (orange line), Dy_3_BB66 (mint line), Dy_3_BB53 (blue line), and Dy_3_BB46 (red line), calculated from the light scattering data. (E) Native PAGE of an equimolar mix of Dy_3_BB55 and Dy_3_BB66 (each protein at 10 μM) and the formation of high-molecular-weight polymers Poly-Dy_3_BB55:66 (lane 3, black arrow). Lanes 1 and 2 represent the mobility of monomer controls Dy_3_BB55 and Dy_3_BB66, respectively. (G) AFM micrograph of Poly-Dy_3_BB55:66. (I) Low-magnification and enlarged (detailed view) ns-TEM view of single-chain filaments (red arrow) and bundled rods (white arrow). (F) Native PAGE of an equimolar mix of Dy_3_BB53 and Dy_3_BB46 (each protein at 10 μM) and the formation of high-molecular-weight polymers Poly-Dy_3_BB53:46 (lane 3, black arrow). Lanes 1 and 2 present the mobility of monomer controls Dy_3_BB53 and Dy_3_BB46, respectively. (H) AFM micrograph of Poly-Dy_3_BB53:46. A detailed view of the branching section is magnified. Entangled structures are marked with white dashed circles. (J) ns-TEM images of the entangled (left image) and long rigid rods (right image, white arrow). A representable single-chain filament is marked with a red arrow.

To mimic the structural arrangement of the central domain of dystrophin, we developed a repertoire of orthogonal bifunctional building blocks based on Dy_3_-neg, each coupled with two identical (Dy_3_BB55 and Dy_3_BB66) or alternate CC-forming peptides (Dy_3_BB53 and Dy_3_BB46) (Figure 4A). We hypothesized that the rigidity of the three spectrin repeats of Dy_3_-neg and tight coupling of CC-forming peptides with the spectrin scaffold should facilitate the formation of extended rods (Poly-Dy_3_BB55:66), rather than closed assemblies (Figures 4B and S9A). Next, we designed two additional building blocks using two pairs of orthogonal CCs (sticky-ends), further mitigating the risk of closed dimer assembly, Dy_3_BB53, and Dy_3_BB46 to form Poly-Dy_3_BB53:46 (Figures 4A and S9B). All purified proteins (Figures S10 and S11) had a high content of α-helical secondary structures aligning with models (Figures 4A and S10, S11), exhibited reversible folding after thermal denaturation (Figures S10 and S11) and were present in the monomeric state (Figure 4C,D and Table S3). Dy_3_BB66 was more stable than Dy_3_BB55 (Figure S10), and similarly, Dy_3_BB46 exhibited higher stability than Dy_3_BB53, indicating the beneficial effect of P6SHb on stability (Figure S11). Overall, the CD spectra and thermal denaturation data support the correct secondary structure, reversible refolding, and high stability of engineered bifunctional protein blocks.

The individual proteins exhibited long-term stability in Tris buffer supplemented with 1 M NaCl. To initiate the head-to-tail self-assembly and polymerization, we mixed both components in an equimolar ratio and reduced the salt concentration to 150 mM. The equimolar mixture of Dy_3_BB55 and Dy_3_BB66 yielded high-molecular-weight protein products (Poly-Dy_3_BB55:66) that were too large to enter the separating part of the PAGE gel, in comparison to monomeric individual building blocks, supporting polymerization through CC-mediated assembly (Figure 4E). Similar results were obtained with an alternate pair comprising two sets of CC-linking peptides, Dy_3_BB53 and Dy_3_BB46, with the products (Poly-Dy_3_BB53:46) again too large to be well-resolved in the PAGE gel (Figure 4F). Additionally, we performed AFM imaging of the central spectrin repeat-based scaffolding unit Dy_3_-neg and utilized bifunctional building blocks, demonstrating the monomeric nature of the individual proteins (Figure S12).

AFM micrographs of Poly-Dy_3_BB55:66 revealed numerous high-molecular-weight assemblies with extended and circular geometries (Figures 4G and S13). AFM image quantification revealed a larger percentage of distinct structures in comparison to amorphous aggregates, highlighting the robustness and efficacy of the self-assembling system (Figures S14 and S15). The resulting polymers revealed end-to-end lengths in the submicrometer range; meanwhile, height profile analysis displayed values of ∼0.7 to 0.8 nm, similar to those measured for dimeric assemblies (Figures 4G and S15, S16). Negative stain transmission electron microscopy (ns-TEM) revealed the presence of up to ∼2 μm long rods, composed of numerous bundled single-chain filaments along the longitudinal axis, achieving a maximum width of ∼35 nm. This corresponds to ∼14 laterally associated filaments. Magnified images displayed several unbundled chains with shorter lengths and a higher degree of flexibility (Figures 4I and S17). AFM micrographs of Poly-Dy_3_BB53:46 unveiled up to ∼6.5 μm long rods, featuring several branching sites (Figures 4H and S18). These images indicate an efficient self-assembly process and strong bundling along the longitudinal axis of individual chains, resulting in enhanced structural reinforcement and efficient polymer growth. In addition, we confirmed the robustness and efficacy by quantifying the AFM images, where we identified a substantial amount of well-defined filamentous structures, which can intertwine into tangles (Figures S19 and S20). Moreover, a height analysis further indicated lateral bundling, resulting in increased height values of ∼1.2 to 1.4 nm (Figures S20 and S21). We observed the coexistence of tortuous single-chain protein filaments with small diameters and thick bundled rods (up to ∼50 nm). This corresponds to bundling of ∼20 single-chain filaments, stacked along the longitudinal axis (Figures 4J and S22).

### Engineering Decorated Building Blocks for Self-Assembly into Functionalized Rods

**Figure 5:**
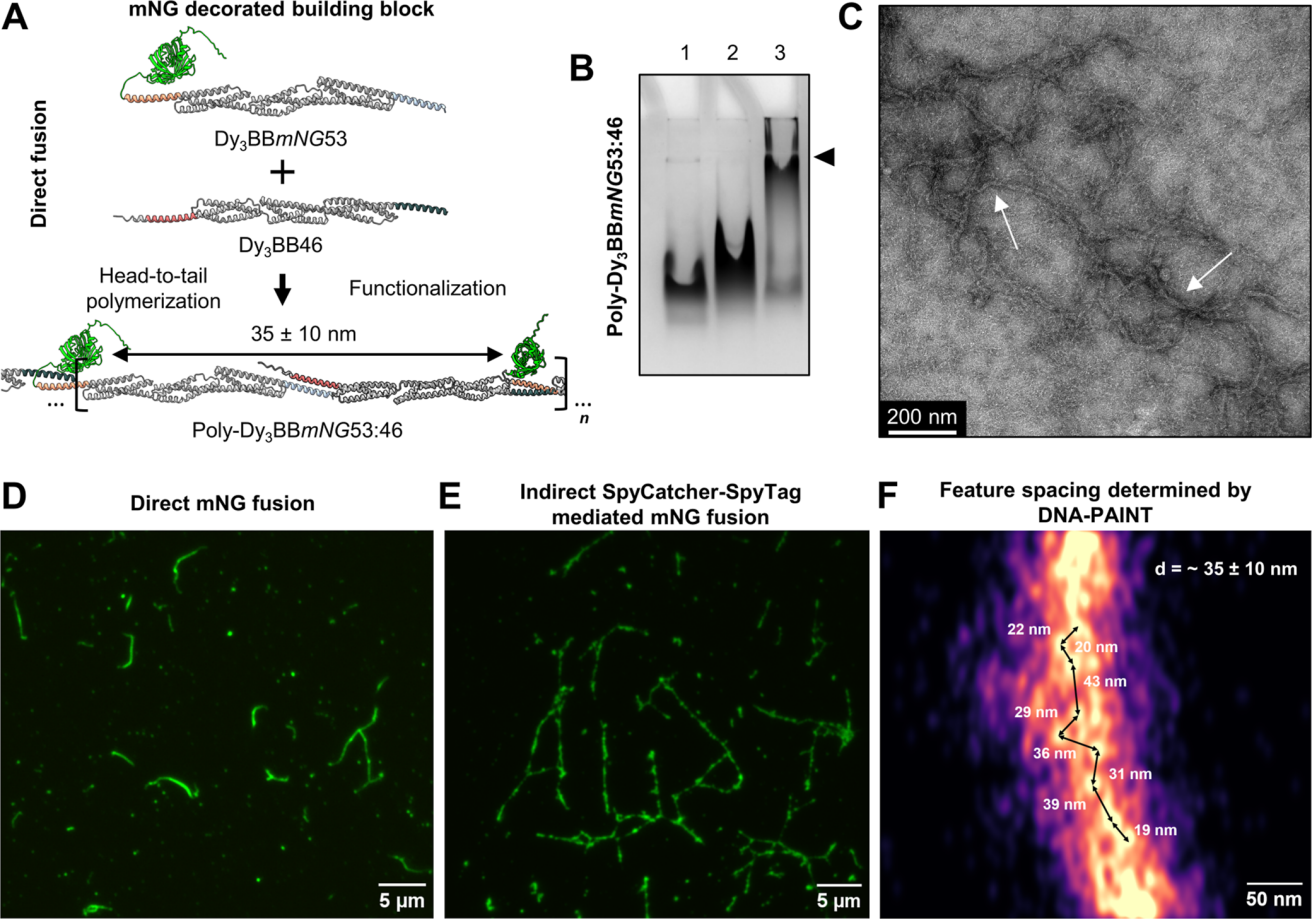
Nanoscale construction of patterned rods functionalized with fluorescent proteins. (A) Protein engineering of the mNeonGreen (mNG)-decorated bifunctional building block (Dy_3_BB*mNG*53) and proposed self-assembly into single-chain filaments Poly-Dy_3_BB*mNG*53:46. The naming convention is similar to before, with a slight difference, where the abbreviation in italics (e.g., *mNG*) denotes the type of fused domain. (B) Native PAGE analysis of resulting high-molecular-weight products Poly-Dy_3_BB*mNG*53:46 (lane 3, black arrow). To control the assembly, lanes 1 and 2 represent the mobility patterns of Dy_3_BB46 and Dy_3_BB*mNG*53 monomers, respectively. (C) ns-TEM image of Poly-Dy_3_BB*mNG*53:46 bundled rods (white arrows). (D,E) TIRFM images of Poly-Dy_3_BB*mNG*53:46 (D) and Poly-Dy_3_BB*spyT*46:53 (E) rods functionalized with mNG. (F) DNA-PAINT super-resolution imaging of Poly-Dy_3_BB*spyT*46:53 rods labeled with the HUHsfGFP_PAINT_-docker oligo construct. Fibers are bundled; nevertheless, individual attachment sites with expected spacing can be resolved (black arrows). The measured distances between displayed proteins (bright dots) are shown next to the corresponding arrows. The expected theoretical distance between the presented proteins is noted in the upper right corner of the image. The separation can range ±10 nm due to the flexible attachment of the decorated protein domains (HUHsfGFP_PAINT_).

Each protein block features freely accessible N- or C-terminal ends, which makes them amenable to genetic fusion with selected functional peptides or domains. To expand the potential of our spectrin repeat-based engineering strategy, we designed two N-terminally decorated bifunctional building blocks, based on Dy_3_BB53 and Dy_3_BB46. This was achieved by directly fusing the mNeonGreen fluorescent protein (mNG) via the flexible linker at the N-terminus, resulting in decorated protein subunits Dy_3_BB*mNG*53 and Dy_3_BB*mNG*46 (Figures 5A and S23A,B). We first tested the system with an equimolar mix (10 μM each protein) of regular (without mNG; Dy_3_BB46) and decorated bifunctional protein blocks (Dy_3_BB*mNG*53). Via this arrangement, mNGs should be spaced by six spectrin domains (∼35 ± 10 nm) after the self-assembly into single-chain filaments (Figure S23A). Molecular models indicated that N-terminal mNG fusion via a flexible GS linker should not impair CC formation (Figures 5A and S23A–C). Proteins expressed in *E. coli* exhibited high purity (Figure S24A) with CD analysis confirming a combination of α-helical and β-barrel characteristics of mNG (Figure S24B–E) and DLS and SEC-MALS confirming the monodispersity and correct size of protein blocks (Figure S24F,G and Table S3).

An equimolar mixture of Dy_3_BB*mNG*53 with Dy_3_BB46 yielded high-molecular-weight products (Poly-Dy_3_BB*mNG*53:46), where the majority was too large to be resolved well in the native PAGE gel as well as some unreacted Dy_3_BB46 (Figure 5B). We observed a similar polymerization pattern when mixing Dy_3_BB*mNG*46 and Dy_3_BB53 (10 μM each protein) (Figure S25A,B). Notably, polymerization did not impair mNG folding, yielding bright green solutions in both cases (Figures S23C and S25C). ns-TEM analysis of Poly-Dy_3_BB*mNG*53:46 exhibited numerous tortuous rods, suggesting a higher degree of flexibility than that observed for Poly-Dy_3_BB53:46 (Figures 5C and S26). The length of polymers spanned well over 1 μm. Similar to those seen for nondecorated polymers, single-chain filaments tended to bundle along the longitudinal axis and intertwine into tangles. This appears to positively affect the stabilization and growth of the filaments. Furthermore, we employed total internal reflection fluorescence microscopy (TIRFM) to confirm the presentation of fluorescent protein domains along the rods. TIRFM revealed well-organized rods up to ∼8 μm in length, exhibiting strong fluorescence signals and demonstrating successful presentation of selected domains (Figures 5D and S27). To improve the ability to functionalize the scaffold, we evaluated the efficiency of protein domain presentation via the SpyCatcher-SpyTag protein–protein ligation system^63^ after polymerization of the Poly-Dy_3_BB*spyT*46:53 rods, which does not interfere with polymerization and bundling. In this arrangement, on average, every second unit in the fiber structure should display a short SpyTag peptide (Dy_3_BBs*pyT*46), amenable to ligation with SpyCatcher fused to mNG (Figure S28A). TIRFM imaging revealed well-defined rods up to 15 μm long, indicating that indirect functionalization led to a higher efficiency of polymerization and a more effective domain display along the fiber (Figures 5E and S28B). Next, we decorated rods simultaneously with two different fluorescent proteins to demonstrate the possibility of coimmobilization of multiple functional proteins without compromising rod morphology (Figure S29A,B). We utilized super-resolution imaging with DNA-PAINT to measure distances between protein domains that decorate the rods. According to the molecular model, these sites should be spaced by six spectrin repeats, resulting in ∼35 ± 10 nm spacing (Figure S30A). This was confirmed by super-resolution fluorescence microscopy using DNA-PAINT, which verified that the domains are indeed separated by the designed distance range (Figure 5F). Moreover, to demonstrate the possibility of adjusting the spacing between the decoration features, we used a mixture of two fluorescent proteins, which extended the average distance between the protein domains of the same type by an additional six spectrin repeats, resulting in ∼75 ± 10 nm spacing between the features decorating the rods (Figure S31A). DNA-PAINT results again confirmed the increased spacing, further validating the suitability of this platform for the spatially organized presentation of various protein or peptide domains in the distance range of tens of nanometers (Figures 5F, S30B, and S31B,C).

### Zn(II)-Regulated Self-Assembly of Spectrin Repeat-Based Rods

**Figure 6:**
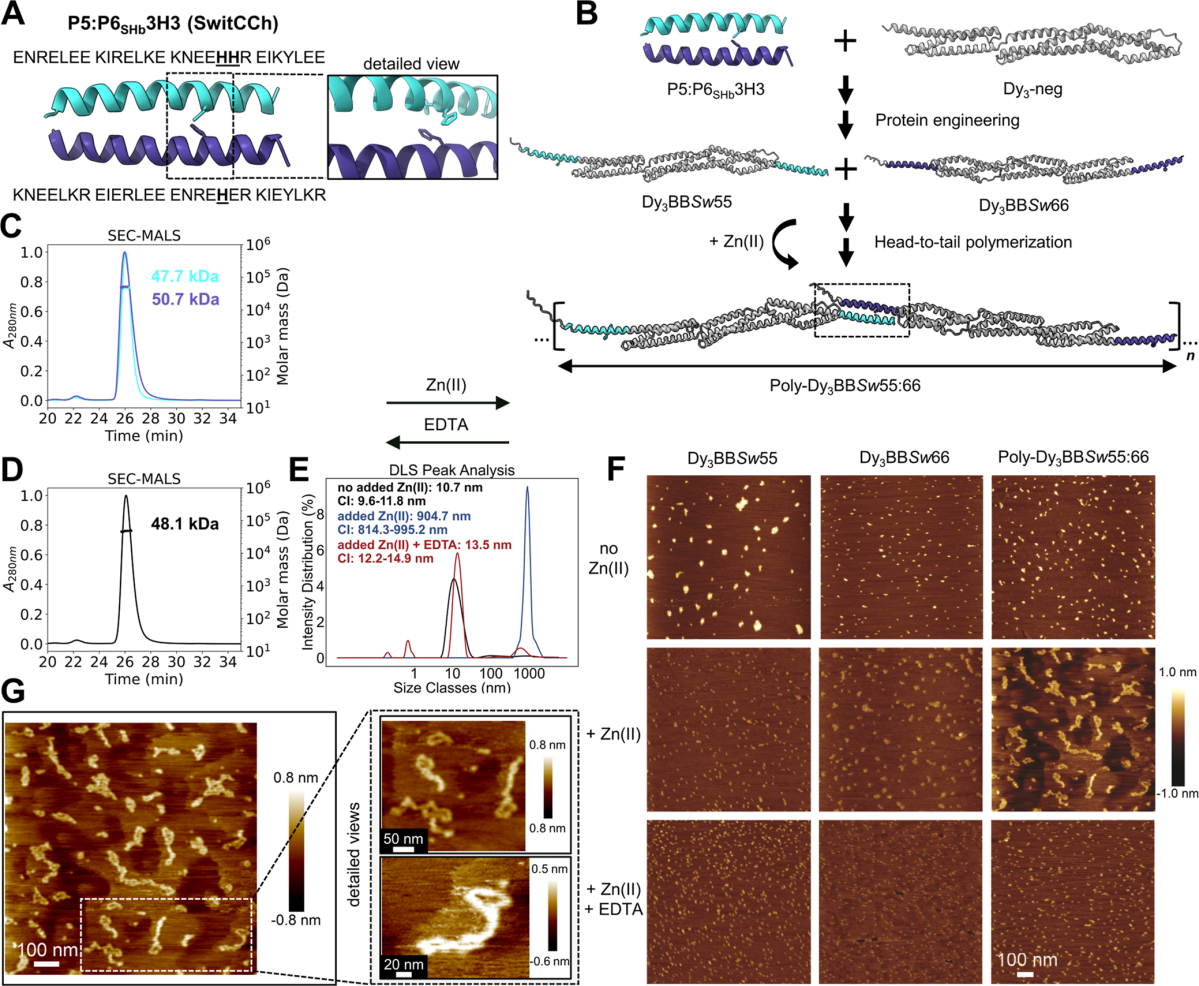
Zn(II)-regulated self-assembly of protein blocks into the rods. (A) Amino acid sequence and molecular model of the P5:P6_SHb_3H3 CC-linking motif. Three His residues form a ZnHis_3_ binding motif (residues are underlined). A detailed view of the ZnHis_3_ motif is shown on the right. (B) Design strategy of the Zn(II)-responsive building blocks. The Dy_3_-neg scaffolding unit is depicted in gray. Peptide P5_SHb_3H3 is depicted in bright blue, and P6_SHb_3H3 is depicted in violet. Upon addition of the molar excess of Zn(II) ions, protein blocks can interact with their binding partner and self-assemble into protein polymers Poly-Dy_3_BB*Sw*55:66. The naming convention is the same as before, except that the abbreviation *Sw* denotes the Zn(II)-responsive CC-linking motif (switCCh). (C,D) SEC-MALS chromatograms with indicated molecular weights of individual protein blocks Dy_3_BB*Sw*55 (bright blue line) and Dy_3_BB*Sw*66 (violet line) and an equimolar mix of both subunits before the addition of Zn(II) ions (15 μM each, black line), calculated from the light scattering data. (E) DLS measurement of high-molecular-weight protein assemblies after mixing both protein blocks (15 μM each) and adding Zn(II) ions (blue line), in comparison to the control mix (no added Zn(II) ions; black line). After adding the EDTA, the peak shifted back to lower size classes (nanometers), corresponding to predominantly monomeric proteins (red line). (F) AFM micrographs of individual protein blocks (Dy_3_BB*Sw*55 and Dy_3_BB*Sw*66) and a mixture of both (Poly-Dy_3_BB*Sw*55:66) in the presence or absence of Zn(II) ions and EDTA. The height profile and size ruler are identical for all images. (G) AFM micrograph of Poly-Dy_3_BB*Sw*55:66 in the presence of Zn(II) ions. A detailed view of selected protein polymers.

Finally, we explored the strategy of reversible regulation of the assembly of spectrin repeat-based filaments by Zn(II) ions. To introduce metal-ion responsiveness into the system, we substituted P5 and P6SHb CCs with Zn-responsive CC variants (peptide pair P5:P6SHb_3H3_, termed SwitCCh),^57^ possessing a designed Zn(II) binding coordination motif made of three His residues (Figure 6A). The two orthogonal bifunctional building blocks (Dy_3_BB*Sw*55 and Dy_3_BB*Sw*66) were produced, isolated, and characterized (Figures 6B,C and S32). To assess the responsiveness of our system to Zn(II) ions, we prepared an equimolar mixture of bifunctional building blocks and analyzed their oligomeric state in the absence of Zn(II) ions using SEC-MALS. As expected, the equimolar mixture of Zn(II)-responsive protein blocks remained monomeric, with an experimentally determined molecular weight of 48.1 kDa, closely matching the theoretical value of 47 kDa (Figure 6D and Table S3). Next, we employed DLS to verify the reversible regulation of the self-assembly process. Upon adding Zn(II) ions, high-molecular-weight products emerged (Poly-Dy_3_BB*Sw*55:66), with an estimated size of ∼900 nm. In contrast, the control mixture with no added Zn(II) was much smaller (∼11 nm). Subsequent addition of ethylenediaminetetraacetic acid (EDTA) triggered the chelation of Zn(II) ions, leading to the disassembly of the high-molecular-weight products and a shift of the peak toward a smaller size. This led to predominantly monomeric proteins matching the initial values of the uninduced mix (Figure 6E).

We conducted an AFM analysis to address the concern that the observed shift toward higher size might be attributed to nonspecific aggregates. Individual protein blocks and the equimolar mixture, designated as Poly-Dy_3_BB*Sw*55:66, remained predominantly monomeric before the addition of Zn(II) ions, aligning closely with the SEC-MALS and DLS results (Figure 6F). The presence of amorphous aggregates in the case of Dy_3_BB*Sw*55 likely resulted from the drying process during AFM sample preparation. Upon combining both protein components and adding Zn(II) ions, we observed numerous filamentous assemblies, closely resembling those obtained throughout the polymerization with SHb CC-forming peptides. The close inspection of visualized rods exhibited end-to-end dimensions of up to ∼320 nm (Figures 6G and S33). Moreover, Poly-Dy_3_BB*Sw*55:66, similarly to Poly-Dy_3_BB55:66, resulted in shorter and less rigid assemblies in the submicrometer range. We supported these observations with image analysis, where quantification revealed a higher percentage of extended structures in comparison to that of amorphous aggregates (Figure S34). Importantly, AFM micrographs showed that Poly-Dy_3_BB*Sw*55:66 had a propensity for lateral bundling, which helped reinforce the single-chain arrangement (Figure 6G). Bundling is likely facilitated by the positively charged nature of Zn(II) ions, engaging in electrostatic interactions with the negatively charged surface residues of Dy_3_-neg.

## Conclusions

Recombinant protein-based biopolymers of high molecular weight offer great potential for the development of designable biomaterials but are often limited by poor expression yields, repetitive sequences, aggregation propensity, and often environmentally hazardous manufacturing processes (e.g., solvent-based wet spinning).^64−68^ Here, we utilized protein engineering to design intrinsically rigid building blocks based on the tandem spectrin repeat originating from the dystrophin central rod domain and designed CC motifs to facilitate the self-assembly into fibrillar structures. The underlying principle of our platform is the engineering of high-expressing, soluble protein blocks, combining the orthogonality of CC-linking segments as well as maintaining the extended arrangement of the core spectrin repeat-based scaffolding domain, which is stabilized by helices rigidly coupling the neighboring units.

Previous investigation of the dystrophin central rod domain indicated an extended, predominantly tortuous single-chain topology with potential actin and microtubule binding sites.^40−42,69,70^ Due to its structural features, we selected a block of three spectrin repeats from the dystrophin region as the rigid scaffolding unit. Modifying the surface residues increased the net negative charge, resulting in high bacterial expression, solubility, and enhanced stabilization. Prior data suggest a slightly tortuous morphology in the region of interest, presumably due to the single polypeptide chain and a lack of external stabilizing factors.^42,69,70^ Several polypeptide-based fibrils have been designed;^71,72^ however, in most cases, the diameter of rods was larger than 10 nm, with monomers annealing laterally,^71^ which limits the ability to decorate these fibrils with proteins by an adjustable separation on the order of tens of nanometers. Extended rigid units, in contrast, allowed spacing of the proteins decorating the fibers in the tens of nanometer range, which is in close agreement with spacings for the most efficient stimulation of B-cell receptors^58^ and, therefore, potentially appropriate for vaccine design. The number of spectrin units and the arrangement of building blocks permit tunable separation from ∼10 to at least 75 nm; hence, they might be suitable for presenting the antigens at the optimal spacing for activation of B-cell receptors. Using orthogonal coupling domains (e.g., SpyTag/SnoopTag, orthogonal CCs) might allow for precise rather than statistical spacing between the presented domains. Additionally, the number of spectrin repeats in each module, e.g., from one to five or more repeats, could be used to adjust the spacing in a wide range, from 10 to >100 nm. Moreover, a more rigid coupling of functional domains could further define the distance range more precisely.

We introduced the self-assembling domains to the scaffolding unit through in-frame terminal fusion with heterodimeric parallel CC peptides with defined orthogonality. The designed parallel heterodimeric SHb peptide pairs exhibited strong affinities in the nanomolar range required for linking spectrin repeats to form large supramolecular assemblies. Molecular modeling provided the appropriate in-frame fusion to maintain the helicity and orientation of neighboring spectrin repeats. Additionally, by using a three-helix bundle fold, we modeled the fusion of parallel CC peptides with an ultrashort helical linker at one or both termini. Importantly, this strategy ensured a seamless extension from the terminal spectrin α-helix to the peptide, reinforcing the structural rigidity at the junction site and preserving the relative orientation of the neighboring spectrin repeat domains. We explored two strategies for extended linear chain assembly based on identical peptides of a single pair on both termini or four peptides originating from two orthogonal pars. Dy_3_BB55 and Dy_3_BB66 yielded filamentous structures, where single chains bundled into rods. ns-TEM imaging revealed bundled rods of over 2 μm in length. These assemblies were reinforced by bundling several single chains into thicker rods along the longitudinal axis. The second orthogonal CC pair was introduced in the bifunctional protein block for alternate peptide arrangement, designated as Dy_3_BB53 and Dy_3_BB46. The improved design with terminal sticky ends resulted in the efficient growth of thick rods, many reaching several micrometers (∼6.5 μm), displaying a well-defined filamentous morphology.

CCs have been used to generate several types of materials, either by chemical conjugates of CC-forming peptides to organic polymers^73,74^ or via genetic fusion to other polypeptides, which formed randomly connected materials (e.g., gels).^75^ Previous attempts also employed CC-mediated polymerization of engineered protein subunits into filamentous structures where β-stranded globular protein domains were combined with helical CC dimers.^54,55^ Here, in contrast, we emphasize matching helicity to increase the rigidity of assemblies and utilizing metal-ion-regulated assembly of CC linkers. Rigid connections between helices of the oligomerizing protein domains have been used before to assemble symmetric protein cages,^76^ which established the requirement to select the appropriate frame of the matching helices. Our results show that the fibrils have some flexibility, which agrees with the observation that helical linkers exhibit slight bending.^77^

The multimicrometer rods (up to 15 μm) enable the decoration and spatially defined presentation of selected proteins, including enzymes (e.g., biocatalytic cascades), antigens for immune response, or biologically active peptides. Previous investigations revealed that natural scaffolding proteins can be exploited for controlled immobilization of enzymes, applicable for the development of one-pot cascade biocatalysis reactions.^78,79^ By comparison, our robust platform provides increased capability for programmable spacing adjustments between immobilization sites along filament chains. While the spacing between decoration features is difficult to adjust on peptide-based supramolecular assemblies,^80,81^ spacing adjustment can be readily achieved in the system presented here through the modification of either the molar ratio of protein modules with different attachment sites, the number of spectrin repeats within individual protein modules, the use of modules with different orthogonal CC coupling pairs, positioning of attachment sites at both ends of the module, or a combination of these strategies.

Zn(II) binding motifs engineered in protein interfaces have been previously employed to regulate the self-assembly of pH-responsive filaments,^82^ finite assemblies like CCPO triangles, and heterodimeric bipyramidal protein cages.^57^ Herein, we simplified the interface design and utilized the P5/P6_SHb_3H3 CC motif in a two-component building block arrangement. While this choice slightly reduced the polymer growth efficiency compared to SHb CC pairs, it offered higher orthogonality and stability than other switCCh CC pairs.^56,57^ The designed system exhibited monodispersity in the absence of Zn(II) ions and facilitated predictable on-target pairing, resulting in the formation of extended rods in the presence of Zn(II) ions. This design underscores the versatility of metal-ion-responsive CC motifs as highly adjustable linking domains, providing control over the assembly process of unbound high-molecular-weight polymers and offering the potential for developing advanced biomaterials with environmentally dependent behavior.

Nature selected spectrin domains to construct several other extended rod-like structures and components of the cytoskeleton, such as spectrin and α-actinin.^83^ α-Actinin, composed of two α-chains, enhances the rigidity of the assembly, suggesting that other spectrin repeat family members or *de novo* designed proteins based on spectrin folds could likely serve as alternative building modules. Moreover, engineering the interaction interfaces between spectrin repeats might potentially replace the CC-mediated assembly and further expand the frontiers of functional protein biomaterials with diverse applications in bionanotechnology and biomedicine.

## Methods

### AF2 Molecular Modeling

Structural models were prepared and tested with AF2 and AlphaFold3.^61,84^ Local installation of Colabfold^85^ version 1.5.2 was used for AlphaFold multimer weights included in version 2.3.0. Multiple sequence alignments were created automatically using the mmseq2 server.^86^ Five models were created for each structure, and the best ranked via a pLDDT score was chosen for further work after a visual inspection. Scripts for running AF2 on a *slurm* cluster are available at https://github.com/ajasja/af2slurm.

### Molecular Cloning

All protein constructs in this study were ordered as synthetic genes from Twist Bioscience (CA, USA) or Integrated DNA Technologies (IA, USA). Genes were designed so that their universal overhangs enabled a simple Gibson assembly (GA) reaction into the amplified pET-41a(+) expression vector. For GA, amplification of the vector backbone was performed with a RepliQa HiFi ToughMix (Quantabio, MA, USA). The polymerase chain reaction was carried out according to the manufacturer’s instructions. Universal primers, used to amplify the vector, are listed in Table S4. Plasmids were constructed using standard protocols of GA.^87^ The amino acid sequences of all cloned proteins are listed in Table S2.

### Protein Expression and Purification

All protein constructs prepared in pET-41a(+) were transformed into an *E. coli* NiCO21 (DE3) production strain (NEB, MA, USA) and grown overnight at 37 °C on LB agar plates supplemented with kanamycin (50 μg/mL). Inoculums were prepared by selecting individual colonies and cultured overnight at 37 °C with agitation at 180 rpm in 100 mL of LB medium supplemented with kanamycin (50 μg/mL). These inoculums were transferred into 5 L fermentation flasks, each containing 1 L of LB media, to achieve a starting optical density (OD) level of 0.1, followed by continued incubation at 37 °C. Upon reaching an OD of ∼0.6, induction was initiated by adding 0.5 mM IPTG (Goldbio, MO, USA). After 18 h of growth at 20 °C, bacterial pellets were collected via centrifugation and stored at −80 °C. Frozen pellets of Dy_3_-neg and monofunctional building blocks from 1 L of fermentation were resuspended on ice in 25 mL of lysis buffer (50 mM Tris pH 8.0, 150 mM NaCl, 10 mM imidazole, 0.5 mM TCEP, 1 mM MgCl_2_, 15 U/mL Benzonase (Merck, Germany)) supplemented with CPI protease inhibitor mix (Millex Sigma-Aldrich, MO, USA). They were then lysed by sonicating (intervals of 1 s ON, 3 s OFF, effective sonication per cycle 1 min, amplitude 60%) for 4 cycles or until the suspension was clarified. Soluble fractions obtained after centrifugation at 16,000*g* (4 °C) for 45 min were filtered through 0.45 μm syringe filters (Minisart, Sartorius, Germany) and added to 6 mL of previously equilibrated Ni-NTA resin on gravity columns. Ni-NTA columns were first washed with buffer A (50 mM Tris pH 8.0, 150 mM NaCl, 10 mM imidazole, 0.5 mM TCEP) for 5 column volumes and B (50 mM Tris pH 8.0, 150 mM NaCl, 30 mM imidazole, 0.5 mM TCEP) until the absorbance at 280 nm reached values below 0.02. The bound fraction was later eluted with the addition of elution buffer (50 mM Tris at pH 8.0, 150 mM NaCl, 0.5 mM TCEP, and 300 mM imidazole). Fractions containing the protein were merged and injected into the size exclusion column (HiLoad 26/600 Superdex 200 pg, GE Healthcare, IL, USA) and separated at 2.5 mL/min flow (20 mM Tris at pH 7.5, 150 mM NaCl, 0.5 mM TCEP, 10% (v/v) glycerol). After the separation, appropriate fractions were merged, concentrated (centrifugal unit 10 or 30 MWCO, Amicon-ultra, Millex Sigma-Aldrich, MO, USA), plunge frozen in liquid nitrogen, and stored at −80 °C. Purity analysis was performed via SDS-PAGE. Notably, the same expression and purification protocols were replicated for all remaining bifunctional building blocks (except Zn(II)-responsive variants), with the important difference of the supplementation of 1 M NaCl in all required buffers (instead of 150 mM). The purification strategy of Zn(II)-responsive bifunctional building blocks (Dy_3_BB*Sw*55 and Dy_3_BB*Sw*66) was slightly modified as 1 mM EDTA disodium salt (Sigma-Aldrich, MO, USA) was added in Ni-NTA and SEC buffer solutions, supplemented with 150 mM NaCl. In the end, Zn(II) ion-free isolated protein solutions were dialyzed three times against the Tris buffer (20 mM Tris pH 7.5, 150 mM NaCl, 0.5 mM TCEP, 10% (v/v) glycerol) for storage at −80 °C.

### Sodium Dodecyl Sulfate Polyacrylamide Gel Electrophoresis

Isolated proteins were analyzed with SDS-PAGE on 10, 12, or 15% polyacrylamide gels. Samples in loading buffer and the prestained ruler for molecular weight were separated on the gel for ∼50 min at 200 V. Visualization was achieved by staining the gel with InstantBlue Coomassie protein stain (Abcam Limited, Cambridge, UK).

### CC-Facilitated Self-Assembly

Mono- and bifunctional protein blocks were isolated as soluble proteins and stored at −80 °C. Monofunctional building blocks were first dialyzed three times against the Tris buffer solution without glycerol (20 mM Tris pH 7.5, 150 mM NaCl, 0.5 mM TCEP) for CC-facilitated dimerization. Next, the dimerization reaction was performed via an equimolar mixture of the corresponding protein components (20 μM of each). The assembly reaction mixture was incubated for at least 1 h at 4 °C before conducting the experiments. Bifunctional building blocks were isolated as soluble monomers and were stable in high salt storage buffer (20 mM Tris (pH 7.5), 1 M NaCl, 0.5 mM TCEP, and 10% (v/v) glycerol). The CC-facilitated polymerization mixture was prepared by an equimolar mix of pairing protein components (10 μM each) in the high salt storage buffer. The polymerization reaction was initiated by three times dialysis of the mixture against the Tris buffer solution supplemented with lower salt concentration and the absence of glycerol (20 mM Tris pH 7.5, 150 mM NaCl, and 0.5 mM TCEP). After the dialysis, the reaction was incubated for an additional 24 h at 4 °C. Zn(II)-responsive bifunctional building blocks (Dy_3_BB*Sw*55 and Dy_3_BB*Sw*66) were first dialyzed three times against the Tris buffer solution without glycerol (20 mM Tris pH 7.5, 150 mM NaCl, and 0.5 mM TCEP). Zn(II)-regulated assembly was achieved by mixing both protein pairs in an equimolar molar ratio (15 μM each). A 40-fold molar excess of ZnCl_2_ (600 μM; Sigma-Aldrich, MO, USA) was added to the equimolar protein mixture to induce fiber formation. The reaction was further incubated for at least 1 h at 4 °C. Before the experiments, polymers were centrifuged for 2 min at 16,000*g*.

### Circular Dichroism

CD spectra were acquired by using a Chirascan CD spectrometer (Applied Photophysics, UK) equipped with a Peltier temperature control system. Peptide and protein samples, dialyzed three times against a phosphate buffer (20 mM sodium phosphate buffer pH 7.4, 0.5 mM TCEP) were prepared at concentrations ranging from 0.15 to 0.3 mg/mL. Measurements were performed within a 1 mm cuvette (Hellma, Germany) spanning the far-UV range (200–280 nm) at 25 °C. CD spectra were recorded with 1 nm increments, a 1 nm bandwidth, and 1 s sampling intervals. Thermal denaturation studies involved heating the samples from 4 to 90 °C at a rate of 1 °C per minute, followed by reverse thermal denaturation, where the samples were cooled from 90 to 4 °C at the same rate. CD signal intensity was monitored at 222 nm. Origin 2018 software (OriginLab Corporation, MA, USA) and the Matplotlib library (Python) were used to process the raw data and plot the experimental values.

### Dynamic Light Scattering

DLS measurements were conducted using a ZetasizerNano (Malvern, UK) at 20 °C with an angle of 173° and a 633 nm laser. The protein size distribution of particles was recorded, and the diameter was calculated using the provided software. Unless otherwise specified, DLS experiments utilized a protein concentration of 15 μM. The central scaffolding module Dy_3_-neg, monofunctional building blocks, and dimers were measured in Tris buffer (20 mM Tris pH 7.5, 150 mM NaCl, and 0.5 mM TCEP) unless otherwise noted. Bifunctional protein blocks were measured in Tris buffer supplemented with a high salt concentration (20 mM Tris pH 7.5, 1 M NaCl, and 0.5 mM TCEP). The Zn(II)-regulated assembly of an equimolar mixture of Dy_3_BB*Sw*55 and Dy_3_BB*Sw*66 (15 μM each) was monitored by adding a 40-fold molar excess of Zn(II) ions to the protein solution (20 mM Tris, pH 7.5, 150 mM NaCl, and 0.5 mM TCEP). Reversibility was assessed by supplementation with an 80-fold molar excess of EDTA disodium salt. Before measurements, all monomer proteins were filtered through 0.1 μm Durapore centrifugal filters (Merck Millipore, MA, USA), except for polymers, which were centrifuged for 2 min at 16,000*g*.

### Isothermal Titration Calorimetry

Synthetic peptides P3, P4, P5, and P6SHb (ProteoGenix, Schiltigheim, France) were dissolved in the Tris buffer (20 mM Tris pH 7.5, 150 mM NaCl, 0.5 mM TCEP), dialyzed three times against a phosphate buffer (20 mM sodium phosphate buffer pH 7.4, 0.5 mM TCEP), degassed, and filtered through 0.1 μm Durapore centrifugal filters (Merck Millipore, MA, USA). The titrations were performed with a Malvern Panalytical (UK) instrument at 25 °C. Solutions containing CC-forming peptides were titrated into the sample cell containing 10 μM of their binding partner, with the peptide corresponding solution being about ten times more concentrated. Raw thermograms were integrated with the software NITPIC,^88^ interaction analysis was performed with SEDPHAT,^89^ and titration curves were plotted with GUSSI software.

### Size Exclusion Chromatography Coupled to Multiangle Light Scattering

SEC-MALS experiments were conducted using a Waters e2695 high-performance liquid chromatography system, which was coupled with a 2489 UV detector (Waters, MA, USA), a Dawn8+ multiple-angle light scattering detector (Wyatt, CA, USA), and a refractive index (RI) detector RI500 (Shodex, Japan). Before analysis, all protein samples, except for polymers (they were not injected into the column due to the size limitations), were filtered using Durapore 0.1 μm centrifuge filters (Merck Millipore, MA, USA). Subsequently, 100 μL of each sample was injected into a Superdex 200 Increase 10/300 GL column (GE Healthcare, IL, USA). Dy_3_-neg, monofunctional, and Zn(II)-responsive bifunctional building blocks were analyzed in a Tris buffer mobile phase (20 mM Tris at pH 7.5, 150 mM NaCl, and 0.5 mM TCEP). In contrast, other bifunctional proteins were measured in a Tris buffer supplemented with 1 M NaCl (20 mM Tris pH 7.5, 1 M NaCl, and 0.5 mM TCEP). Data analysis was performed using Astra 7.0 software (Wyatt, CA, USA), with the RI signal utilized as the concentration source. SEC-MALS chromatograms were generated using the Matplotlib library in Python.

### Native Polyacrylamide Gel Electrophoresis

To validate the CC-facilitated self-assembly, ∼6 μg samples of the monomer, dimer, or polymer were loaded into separate wells. The native PAGE experiment was conducted using the miniPROTEAN apparatus (Bio-Rad, CA, USA) with a 6 or 8% (w/v) discontinuous polyacrylamide gel (pH 8.8) in cold electrophoresis buffer (25 mM Tris–HCl pH 8.3 and 192 mM glycine) at 90 V for ∼3.5 h. The loading buffer was prepared without SDS. After electrophoresis, the gels were stained with InstantBlue Coomassie protein stain (Abcam Limited, Cambridge, UK) and subsequently scanned for analysis.

### Small-Angle X-ray Scattering Measurements

SAXS measurements of the scaffolding unit Dy_3_-neg were performed at the P12 beamline of PETRA III-DESY (Hamburg, Germany).^90^ We measured in the batch mode using a robotic sample changer in the flow-through mode and utilized an X-ray wavelength of 1.24 Å and a Pilatus 6 M detector that was positioned at a distance of 3 m from the sample. The scattering vector was recorded in the range 0.028–7.3 nm^–1^. We used a dilution series (22.5, 11.25, 5.63, 2.8, and 1.4 mg/mL) to evaluate the effects of concentration. Each dilution sample was recorded with 40 exposure frames, each 0.1 s long. The frames that did not exhibit radiation damage were then averaged and integrated into the SASFLOW pipeline.^90,91^ Between each dilution sample, buffer scattering data were collected for background subtraction purposes. We performed the analysis of scattering curves and ab initio modeling using the ATSAS suite^91^ and used Pepsi-SAXS^92^ to calculate the theoretical SAXS profiles from molecular models and to compare them to experimental data. The agreement between theoretical and experimental curves was assessed by utilizing the χ criterion, where lower scores indicate a stronger correspondence. Models exhibiting the closest agreement with experimental data underwent further enhancement using the online refinement tool Sreflex^93^ from the ATSAS suite,^94^ enabling flexible adjustments to enhance the alignment further.

### Atomic Force Microscopy

Images were obtained with a Nanoscope IIIa Multimode scanning probe microscope (Digital Instruments, Santa Barbara, USA) operating in the tapping mode. Protein samples were centrifuged for 2 min at 16,000*g*. Fifteen microliters of diluted protein solution (∼0.01 μM) was spread across freshly cleaved mica surface and incubated for 8 min at room temperature. After incubation, the sample was gently washed with pure water and dried with a stream of nitrogen. Standard tapping mode probes from an OTESPA (Mikromasch) with a tip radius below 7 nm and a nominal resonant frequency of 300 kHz were used. Images were taken at the scan rate of 1 Hz, and the image resolution was 512 × 512 pixels. Raw images were processed with NanoScope Analysis software (Bruker, MA, USA). The analysis of processed images was performed with the automatic morphology quantification method NeurphologyJ and particle analysis tools in Fiji.^95,96^ Length analysis was conducted by skeleton analysis in Fiji; meanwhile, height analysis of micrographs was performed with the Gwyddion software (Czech Metrology Institute, Czech Republic).

### Transmission Electron Microscopy

Diluted protein samples (∼0.5 μM) were mixed gently, applied on freshly glow-discharged copper grids (400 mesh, Formvar-carbon coated), washed with pure water, and stained with one droplet of 1% (w/v) water solution of uranyl acetate for 3 s. The grids were observed by a transmission electron microscope TALOS L120C (ThermoFischer SCIENTIFIC, The Netherlands), operating at 100 kV, and micrographs were recorded with a Ceta 16 M camera using Velox software.

### Total Internal Reflection Fluorescence Microscopy

Six channel Ibidi 1.5H glass coverslip slides (cat. 80607) were cleaned using 2% Hellmanex (Sigma-Aldrich, MO, USA, cat. Z805939-1EA) solution and 10 M KOH. Next, 50 μL of PLL-*g*-PEG (SuSoS AG, Switzerland, cat. NB02-43) at a concentration of 0.3 mg/mL was flushed into the chamber, incubated for 30 min, and then washed with Tris buffer (20 mM Tris pH 7.5, 150 mM NaCl, 0.5 mM TCEP). Fibers were diluted to a concentration of ∼2 μM and deposited on the slides for 1 h.

### Super-Resolution Microscopy with DNA-PAINT

The fibers were labeled with a HUHsfGFP_PAINT_ construct, which was attached to a 5′-AAG TAT TAC CAG TCC TCC TCC TCC TCC TCC TCC TCC TCC TCC T-3′ docking DNA strand (docker oligo) ordered from Eurofins Genomics (Germany). The first part of the DNA was needed for the attachment to HUH, and 5′-TCC TCC TCC TCC TCC TCC T-3′ contains five repeats of the R1 docking strand.^97^ The HUHsfGFP_PAINT_ construct and the docker oligo were mixed in a 1:1 molar ratio in the reaction buffer (50 mM *N*-(2-hydroxyethyl)piperazine-*N*′-ethanesulfonic acid, 50 mM NaCl, 1 mM MgCl_2_, 1 mM MnCl_2_, pH 8) and incubated at 37 °C for 3 h (following the protocol of Klaus et al.).^98^ The conjugated construct was then purified with SEC using a Superdex 200 Increase 10/300 GL column (GE Healthcare, IL, USA). The design of the docking and imaging strand R1 (5′-AGGAGGA-3′) was taken from Strauss and Jungmann.^97^

Six channel Ibidi 1.5H glass coverslip slides (cat. 80607) were cleaned with 2% Hellmanex solution (Sigma-Aldrich, MO, USA, cat. Z805939-1EA) and 10 M KOH. First, 50 μL of PLL-*g*-PEG (SuSoS AG, Switzerland, cat. NB02-43) at a concentration of 0.3 mg/mL was flushed into the chamber, incubated for 30 min, and then washed with Tris buffer (20 mM Tris pH 7.5, 150 mM NaCl, 0.5 mM TCEP). Fibers were incubated with a HUHsfGFP_PAINT_-docker oligo construct in a 1:1 molar ratio and incubated at room temperature for 30 min. The fibers were then diluted to a concentration of ∼2 μM and deposited on the slides for 1 h.

The imaging strand 5′-Atto 655-AGG AGG A-3′ (Metabion, Germany) was added in 1 nM final concentration to a Tris buffer (20 mM Tris pH 7.5, 150 mM NaCl, 0.5 mM TCEP) supplemented with an oxygen scavenger mixture (108500 U/mL catalase from bovine liver (Sigma-Aldrich, MO, USA, cat. C40)), 8250 U/mL glucose oxidase (Sigma-Aldrich, MO, USA, cat. G7141), 0.65% glucose (Sigma-Aldrich, MO, USA, cat. G5767), and trollox (Sigma-Aldrich, MO, USA, cat. 238813) at a concentration of 2 mg/mL.

DNA-PAINT was performed with the same microscope setup as that used for TIRF imaging.^99^ The imager was excited at 638 nm at 50% laser power (3.7 mW), and a 680/420 emission filter was used. Camera integration time was 100 ms. The effective pixel size was 72 nm. DNA paint images were processed using Picasso 0.6.1.^99^ A box size of 7 pixels and a gradient of 1500 pixels were used for localization. Localizations were drift-corrected by using the built-in RCC drift correction.

## Supporting Information Available

Amino acid sequences of synthetic peptides used in the study; annotated amino acid sequences and net charge values at neutral pH of proteins used in the study; SEC-MALS measurements of isolated protein constructs; list of primers used for GA; protein engineering and structural analysis of the spectrin repeat-based scaffolding unit; biophysical characterization of Dy_3_-neg; SAXS structural characterization of Dy_3_-neg; structural features of the protein block design; CC stabilization via electrostatic interactions; purification analysis and biophysical characterization of Dy_3_BB3 and Dy_3_BB4; purification analysis and biophysical characterization of Dy_3_BB6 and Dy_3_BB5; DLS and AFM characterization of CC-facilitated dimer assembly; schematic explanation of bifunctional building block design and proposed mechanism of head-to-tail polymerization; purification analysis and biophysical characterization of Dy_3_BB55 and Dy_3_BB66; purification analysis and biophysical characterization of Dy_3_BB53 and Dy_3_BB46; AFM imaging of individual proteins; AFM imaging of Poly-Dy_3_BB55:66; image quantification of Poly-Dy_3_BB55:66; particle analysis of Poly-Dy_3_BB55:66; morphology analysis of Poly-Dy_3_BB55:66; ns-TEM imaging of Poly-Dy_3_BB55:66; AFM imaging of Poly-Dy_3_BB53:46; image quantification of Poly-Dy_3_BB53:46; particle analysis of Poly-Dy_3_BB53:46; morphology analysis of Poly-Dy_3_BB53:46; ns-TEM imaging of Poly-Dy_3_BB53:46; self-assembly of functionalized rods Poly-Dy_3_BB*mNG*53:46; purification analysis and biophysical characterization of Dy_3_BB*mNG*53 and Dy_3_BB*mNG*46; self-assembly of functionalized rods Poly-Dy_3_BB*mNG*46:53; ns-TEM images of Poly-Dy_3_BB*mNG*53:46; TIRFM images of Poly-Dy_3_BB*mNG*53:46; indirect functionalization and TIRFM imaging of Poly-Dy_3_BB*spyT*46:53 rods; co-immobilization of two fluorescence proteins on Poly-Dy_3_BB*spyT*46:53 rods; super-resolution imaging with DNA-PAINT; super-resolution imaging of coimmobilized rods with DNA-PAINT; purification analysis and biochemical characterization of Dy_3_BB*Sw*55 and Dy_3_BB*Sw*66; morphology analysis of Poly-Dy_3_BB*Sw*55:66; and image quantification of Poly-Dy_3_BB*Sw*55:66 (DOCX)

## Abbreviations

CC: coiled-coil
ABD1: N-terminal actin-binding domain
CRD: cysteine-rich domain
CTD: C-terminal binding domain
CCPO: coiled-coil protein origami
AF2: AlphaFold2
Ni-NTA: Ni^2+^ coupled to nitrilotriacetic acid
SEC: size exclusion chromatography
SAXS: small-angle X-ray scattering
ITC: isothermal titration calorimetry
DLS: dynamic light scattering
CD: circular dichroism
SEC-MALS: size exclusion chromatography coupled to multiangle light scattering
AFM: atomic force microscopy
ns-TEM: negative stain transmission electron microscopy
mNG: mNeonGreen fluorescent protein
TIRFM: total internal reflection fluorescence microscopy
