# Supplementary material for: Coupling of Spectrin Repeat Modules for the Assembly of Nanorods and Presentation of Protein Domains
Source: ACS Nano. 2024 Oct 11;18(42):28748–63. doi: 10.1021/acsnano.4c07701 (PMC11503911; doi:10.1021/acsnano.4c07701)
Supplement: Supplementary file 1 — nn4c07701_si_001.docx [file nn4c07701_si_001.docx]

Supporting Information

for

# **Coupling of Spectrin Repeat Modules for the Assembly of Nanorods and Presentation of Protein Domains**

**Short title:** Coupling of Spectrin Repeat Modules for Nanorod Assembly

Klemen Mezgec*^1,2^, Jaka Snoj^1,2^, Liza Ulčakar^1,2^, Ajasja Ljubetič^1,3^, Magda Tušek Žnidarič^4^, Miha Škarabot^5^, Roman Jerala^1,6🖂^

^1^ Department of Synthetic Biology and Immunology, National Institute of Chemistry, SI-1000 Ljubljana, Slovenia.

^2^ Graduate School of Biomedicine, University of Ljubljana, SI-1000 Ljubljana, Slovenia.

^3^ EN-FIST Centre of Excellence, SI-1000 Ljubljana, Slovenia.

^4^ [Department of Biotechnology and Systems Biology](http://atpscan.global.hornetsecurity.com/index.php?atp_str=hcbq9XbThTo4-IRtSOeEVhWA9WcpOtPL3vSNqQdVdQuZOm8aJAASzBnVx_EvVwWzLWTY2RMWVNypEwYl1OtPyPuAwUxRz8-lCQThxQN3mv_0drW55xIMlUlnTQhrqVjr-1a9vLknlocnQ_gggDB5wRnWRxSwDfcBQrtejMU3tSt1X9HpQsskKA1LD1MzirJHVMRkDZXCd3wknu9S8oXOGtI4koTur95BECFLriujpYR_VKFHtdfFLNJje4Q0wJPbY7-_LRfk14PxML4l1TBlO0VIqEpKu-IXyOSszPOTaIhEycQusq0CRNOGVeEHVfdKyCAZ_4N_hW2LWqEuEu4NJoLt7UdjtJkHdoP4Izo6I5sVfTU-H0FlaMFvjiM6OiPknWjtqYEJ8VNh0gQY-Wp8), National Institute of Biology, SI-1000 Ljubljana, Slovenia.

^5^ Condensed Matter Department, Jozef Stefan Institute, SI-1000 Ljubljana, Slovenia.

^6^ CTGCT, Centre of Technology of Gene and Cell Therapy, Hajdrihova 19, SI-1000 Ljubljana, Slovenia

* First author

^🖂^ Email: [roman.jerala@ki.si](mailto:roman.jerala@ki.si)

**This supplementary information includes:**

- Tables S1-S4
- Figures S1-S34

**Supporting information**

[**Table S1:** Amino acid sequences of synthetic peptides used in the study.	3](#_Toc168408472)

[**Table S2:** Annotated amino acid sequences and net charge values at neutral pH of proteins used in the study.	3](#_Toc168408473)

[**Table S3:** SEC-MALS measurements of isolated protein constructs.	6](#_Toc168408474)

[**Table S4:** List of primers used for Gibson assembly (GA).	7](#_Toc168408475)

[**Figure S1:** Protein engineering and structural analysis of the spectrin repeat-based scaffolding unit.	8](#_Toc174452097)

[**Figure S2:** Biophysical characterization of the Dy_3_-neg.	9](#_Toc174452098)

[**Figure S3:** SAXS structural characterization of the Dy_3_-neg.	10](#_Toc174452099)

[**Figure S4:** Structural features of the protein block design.	11](#_Toc174452100)

[**Figure S5:** Coiled-coil (CC) stabilization via electrostatic interactions.	12](#_Toc174452101)

[**Figure S6:** Purification analysis and biophysical characterization of Dy_3_BB3 and Dy_3_BB4.	13](#_Toc174452102)

[**Figure S7:** Purification analysis and biophysical characterization of Dy_3_BB6 and Dy_3_BB5.	14](#_Toc174452103)

[**Figure S8:** DLS and AFM characterization of CC-facilitated dimer assembly.	15](#_Toc174452104)

[**Figure S9:** Schematic explanation of bifunctional building block design and proposed mechanism of head-to-tail polymerization.	16](#_Toc174452105)

[**Figure S10:** Purification analysis and biophysical characterization of Dy_3_BB55 and Dy_3_BB66.	17](#_Toc174452106)

[**Figure S11:** Purification analysis and biophysical characterization of Dy_3_BB53 and Dy_3_BB46.	18](#_Toc174452107)

[**Figure S12:** AFM imaging of individual proteins.	19](#_Toc174452108)

[**Figure S13:** AFM imaging of Poly-Dy_3_BB55:66.	20](#_Toc174452109)

[**Figure S14:** Image quantification of Poly-Dy_3_BB55:66.	21](#_Toc174452110)

[**Figure S15:** Particle analysis of Poly-Dy_3_BB55:66.	22](#_Toc174452111)

[**Figure S16:** Morphology analysis of Poly-Dy_3_BB55:66.	23](#_Toc174452112)

[**Figure S17:** Negative stain TEM imaging of Poly-Dy_3_BB55:66.	24](#_Toc174452113)

[**Figure S18:** AFM imaging of Poly-Dy_3_BB53:46.	25](#_Toc174452114)

[**Figure S19:** Image quantification of Poly-Dy_3_BB53:46.	26](#_Toc174452115)

[**Figure S20:** Particle analysis of Poly-Dy_3_BB53:46.	28](#_Toc174452116)

[**Figure S21:** Morphology analysis of Poly-Dy_3_BB53:46.	29](#_Toc174452117)

[**Figure S22:** Negative stain TEM imaging of Poly-Dy_3_BB53:46.	30](#_Toc174452118)

[**Figure S23:** Self-assembly of functionalized rods Poly-Dy_3_BB*mNG*53:46.	31](#_Toc174452119)

[**Figure S24:** Purification analysis and biophysical characterization of Dy_3_BB*mNG*53 and Dy_3_BB*mNG*46.	32](#_Toc174452120)

[**Figure S25:** Self-assembly of functionalized rods Poly-Dy_3_BB*mNG*46:53.	33](#_Toc174452121)

[**Figure S26:** Negative stain TEM images of Poly-Dy_3_BB*mNG*53:46.	34](#_Toc174452122)

[**Figure S27:** TIRFM images of Poly-Dy_3_BB*mNG*53:46.	35](#_Toc174452123)

[**Figure S28:** Indirect functionalization and TIRFM imaging of Poly-Dy_3_BB*spyT*46:53 rods.	36](#_Toc174452124)

[**Figure S29:** Co-immobilization of two fluorescence proteins on Poly-Dy_3_BB*spyT*46:53 rods.	37](#_Toc174452125)

[**Figure S30:** Super-resolution imaging with DNA-PAINT.	38](#_Toc174452126)

[**Figure S31:** Super-resolution imaging of co-immobilized rods with DNA-PAINT.	39](#_Toc174452127)

[**Figure S32:** Purification analysis and biochemical characterization of Dy_3_BB*Sw*55 and Dy_3_BB*Sw*66.	41](#_Toc174452128)

[**Figure S33:** Morphology analysis of Poly-Dy_3_BB*Sw*55:66.	42](#_Toc174452129)

[**Figure S34:** Image quantification of Poly-Dy_3_BB*Sw*55:66.	43](#_Toc174452130)

**Table S1:** **Amino acid sequences of synthetic peptides used in the study.**

| **Peptide name** | **Amino acid sequence**  ***gabcdef gabcdef gabcdef gabcdef*** |
| --- | --- |
| **P3SHb** | EIKELEE EIRELEE KNEELKR KNEWLKR GC |
| **P4SHb** | KIEELKR KIEKLKR ENERLER ENEWLER GC |
| **P5SHb** | ENRELEE KIRELKE KNEELKR EIKYLEE GC |
| **P6SHb** | KNEELKR EIERLEE ENRELER KIEYLKR GC |

**Table S2:** **Annotated amino acid sequences and net charge values at neutral pH of proteins used in the study.** Protein segments are marked with specific colors and linker sequences are underlined.

| **Protein name** | **Amino acid sequences of designed protein constructs** | **Net charge** |
| --- | --- | --- |
| **Dy_3_-neg** | Segments in order: **HisTag**-Dy_3_  M**HHHHHHHH**GSGIIAFYNQLQQLEQMTTTAENWLKIQPTTPSEPTAIKSQLKICKDEVNRLSDLQPQIERLKIQSIALKEKGQGPMFLDADFVAFTNHFKQVFSDVQAREKELQTIFDTLPPMRYQETMSAIRTWVQQSETKLSIPQLSVTDYEIMEQRLEELQALQSSLQEQQSELYYLSTTVKEMSKKAPSEISRKYQSEFEEIEERWKKLSSQLVEHCQKLEEQMNKLRKIQNHIQTLKKWMAEVDVFLKEEWPALGDSEILKKQLKQCRLLVSDIQTIQPSLNSVNEEGQKIKNEAEPEFASRLETELKELNTQWDHMCQQVYARKEALKEEL | −10.7 |
| **Dy_3_BB3** | Segments in order: **HisTag**-Dy_3_-P3SHb  M**HHHHHHHH**GSGIIAFYNQLQQLEQMTTTAENWLKIQPTTPSEPTAIKSQLKICKDEVNRLSDLQPQIERLKIQSIALKEKGQGPMFLDADFVAFTNHFKQVFSDVQAREKELQTIFDTLPPMRYQETMSAIRTWVQQSETKLSIPQLSVTDYEIMEQRLEELQALQSSLQEQQSELYYLSTTVKEMSKKAPSEISRKYQSEFEEIEERWKKLSSQLVEHCQKLEEQMNKLRKIQNHIQTLKKWMAEVDVFLKEEWPALGDSEILKKQLKQCRLLVSDIQTIQPSLNSVNEEGQKIKNEAEPEFASRLETELKELNTQWDHMCQQVYARKEALKEELEEIKELEEEIRELEEKNEELKRKNEWLKR | −14.7 |
| **Dy_3_BB4** | Segments in order: P4SHb-Dy_3_-**HisTag**  MKIEELKRKIEKLKRENERLERENEWLEREIIAFYNQLQQLEQMTTTAENWLKIQPTTPSEPTAIKSQLKICKDEVNRLSDLQPQIERLKIQSIALKEKGQGPMFLDADFVAFTNHFKQVFSDVQAREKELQTIFDTLPPMRYQETMSAIRTWVQQSETKLSIPQLSVTDYEIMEQRLEELQALQSSLQEQQSELYYLSTTVKEMSKKAPSEISRKYQSEFEEIEERWKKLSSQLVEHCQKLEEQMNKLRKIQNHIQTLKKWMAEVDVFLKEEWPALGDSEILKKQLKQCRLLVSDIQTIQPSLNSVNEEGQKIKNEAEPEFASRLETELKELNTQWDHMCQQVYARKEALKEELGSG**HHHHHHHH** | −10.6 |
| **Dy_3_BB6** | Segments in order: **HisTag**-Dy_3_-P6SHb  M**HHHHHHHH**GSGIIAFYNQLQQLEQMTTTAENWLKIQPTTPSEPTAIKSQLKICKDEVNRLSDLQPQIERLKIQSIALKEKGQGPMFLDADFVAFTNHFKQVFSDVQAREKELQTIFDTLPPMRYQETMSAIRTWVQQSETKLSIPQLSVTDYEIMEQRLEELQALQSSLQEQQSELYYLSTTVKEMSKKAPSEISRKYQSEFEEIEERWKKLSSQLVEHCQKLEEQMNKLRKIQNHIQTLKKWMAEVDVFLKEEWPALGDSEILKKQLKQCRLLVSDIQTIQPSLNSVNEEGQKIKNEAEPEFASRLETELKELNTQWDHMCQQVYARKEALKEELEKNEELKREIERLEEENRELERKIEYLKR | −12.7 |
| **Dy_3_BB5** | Segments in order: P5SHb-Dy_3_-**HisTag**  MENRELEEKIRELKEKNEELKREIKYLEEEIIAFYNQLQQLEQMTTTAENWLKIQPTTPSEPTAIKSQLKICKDEVNRLSDLQPQIERLKIQSIALKEKGQGPMFLDADFVAFTNHFKQVFSDVQAREKELQTIFDTLPPMRYQETMSAIRTWVQQSETKLSIPQLSVTDYEIMEQRLEELQALQSSLQEQQSELYYLSTTVKEMSKKAPSEISRKYQSEFEEIEERWKKLSSQLVEHCQKLEEQMNKLRKIQNHIQTLKKWMAEVDVFLKEEWPALGDSEILKKQLKQCRLLVSDIQTIQPSLNSVNEEGQKIKNEAEPEFASRLETELKELNTQWDHMCQQVYARKEALKEELGSG**HHHHHHHH** | −14.6 |
| **Dy_3_BB55** | Segments in order: **HisTag**-P5SHb-Dy_3_-P5SHb  M**HHHHHHHH**GSGENRELEEKIRELKEKNEELKREIKYLEEEIIAFYNQLQQLEQMTTTAENWLKIQPTTPSEPTAIKSQLKICKDEVNRLSDLQPQIERLKIQSIALKEKGQGPMFLDADFVAFTNHFKQVFSDVQAREKELQTIFDTLPPMRYQETMSAIRTWVQQSETKLSIPQLSVTDYEIMEQRLEELQALQSSLQEQQSELYYLSTTVKEMSKKAPSEISRKYQSEFEEIEERWKKLSSQLVEHCQKLEEQMNKLRKIQNHIQTLKKWMAEVDVFLKEEWPALGDSEILKKQLKQCRLLVSDIQTIQPSLNSVNEEGQKIKNEAEPEFASRLETELKELNTQWDHMCQQVYARKEALKEELEENRELEEKIRELKEKNEELKREIKYLEE | −18.8 |
| **Dy_3_BB66** | Segments in order: **HisTag**-P6SHb-Dy_3_-P6SHb  M**HHHHHHHH**GSGKNEELKREIERLEEENRELERKIEYLKREIIAFYNQLQQLEQMTTTAENWLKIQPTTPSEPTAIKSQLKICKDEVNRLSDLQPQIERLKIQSIALKEKGQGPMFLDADFVAFTNHFKQVFSDVQAREKELQTIFDTLPPMRYQETMSAIRTWVQQSETKLSIPQLSVTDYEIMEQRLEELQALQSSLQEQQSELYYLSTTVKEMSKKAPSEISRKYQSEFEEIEERWKKLSSQLVEHCQKLEEQMNKLRKIQNHIQTLKKWMAEVDVFLKEEWPALGDSEILKKQLKQCRLLVSDIQTIQPSLNSVNEEGQKIKNEAEPEFASRLETELKELNTQWDHMCQQVYARKEALKEELEKNEELKREIERLEEENRELERKIEYLKR | −14.8 |
| **Dy_3_BB53** | Segments in order: **HisTag**-P5Shb-Dy_3_-P3SHb  M**HHHHHHHH**GSGENRELEEKIRELKEKNEELKREIKYLEEEIIAFYNQLQQLEQMTTTAENWLKIQPTTPSEPTAIKSQLKICKDEVNRLSDLQPQIERLKIQSIALKEKGQGPMFLDADFVAFTNHFKQVFSDVQAREKELQTIFDTLPPMRYQETMSAIRTWVQQSETKLSIPQLSVTDYEIMEQRLEELQALQSSLQEQQSELYYLSTTVKEMSKKAPSEISRKYQSEFEEIEERWKKLSSQLVEHCQKLEEQMNKLRKIQNHIQTLKKWMAEVDVFLKEEWPALGDSEILKKQLKQCRLLVSDIQTIQPSLNSVNEEGQKIKNEAEPEFASRLETELKELNTQWDHMCQQVYARKEALKEELEEIKELEEEIRELEEKNEELKRKNEWLKR | −18.8 |
| **Dy_3_BB46** | Segments in order: **HisTag**-P4SHb-Dy_3_-P6Shb  M**HHHHHHHH**GSGKIEELKRKIEKLKRENERLERENEWLEREIIAFYNQLQQLEQMTTTAENWLKIQPTTPSEPTAIKSQLKICKDEVNRLSDLQPQIERLKIQSIALKEKGQGPMFLDADFVAFTNHFKQVFSDVQAREKELQTIFDTLPPMRYQETMSAIRTWVQQSETKLSIPQLSVTDYEIMEQRLEELQALQSSLQEQQSELYYLSTTVKEMSKKAPSEISRKYQSEFEEIEERWKKLSSQLVEHCQKLEEQMNKLRKIQNHIQTLKKWMAEVDVFLKEEWPALGDSEILKKQLKQCRLLVSDIQTIQPSLNSVNEEGQKIKNEAEPEFASRLETELKELNTQWDHMCQQVYARKEALKEELEKNEELKREIERLEEENRELERKIEYLKR | −12.8 |
| **Dy_3_BB*mNG*53** | Segments in order: **HisTag**-mNG-P5SHb-Dy_3_-P3SHb  M**HHHHHHHH**GSGVSKGEEDNMASLPATHELHIFGSINGVDFDMVGQGTGNPNDGYEELNLKSTKGDLQFSPWILVPHIGYGFHQYLPYPDGMSPFQAAMVDGSGYQVHRTMQFEDGASLTVNYRYTYEGSHIKGEAQVKGTGFPADGPVMTNSLTAADWSRSKKTYPNDKTIISTFKWSYTTGNGKRYRSTARTTYTFAKPMAANYLKNQPMYVFRKTELKHSKTELNFKEWQKAFTDVMGMDELYKGGSGGGSGENRELEEKIRELKEKNEELKREIKYLEEEIIAFYNQLQQLEQMTTTAENWLKIQPTTPSEPTAIKSQLKICKDEVNRLSDLQPQIERLKIQSIALKEKGQGPMFLDADFVAFTNHFKQVFSDVQAREKELQTIFDTLPPMRYQETMSAIRTWVQQSETKLSIPQLSVTDYEIMEQRLEELQALQSSLQEQQSELYYLSTTVKEMSKKAPSEISRKYQSEFEEIEERWKKLSSQLVEHCQKLEEQMNKLRKIQNHIQTLKKWMAEVDVFLKEEWPALGDSEILKKQLKQCRLLVSDIQTIQPSLNSVNEEGQKIKNEAEPEFASRLETELKELNTQWDHMCQQVYARKEALKEELEEIKELEEEIRELEEKNEELKRKNEWLKR | −18.5 |
| **Dy_3_BB*mNG*46** | Segments in order: **HisTag**-mNG-P4SHb-Dy_3_-P6SHb  M**HHHHHHHH**GSGVSKGEEDNMASLPATHELHIFGSINGVDFDMVGQGTGNPNDGYEELNLKSTKGDLQFSPWILVPHIGYGFHQYLPYPDGMSPFQAAMVDGSGYQVHRTMQFEDGASLTVNYRYTYEGSHIKGEAQVKGTGFPADGPVMTNSLTAADWSRSKKTYPNDKTIISTFKWSYTTGNGKRYRSTARTTYTFAKPMAANYLKNQPMYVFRKTELKHSKTELNFKEWQKAFTDVMGMDELYKGGSGGGSGKIEELKRKIEKLKRENERLERENEWLEREIIAFYNQLQQLEQMTTTAENWLKIQPTTPSEPTAIKSQLKICKDEVNRLSDLQPQIERLKIQSIALKEKGQGPMFLDADFVAFTNHFKQVFSDVQAREKELQTIFDTLPPMRYQETMSAIRTWVQQSETKLSIPQLSVTDYEIMEQRLEELQALQSSLQEQQSELYYLSTTVKEMSKKAPSEISRKYQSEFEEIEERWKKLSSQLVEHCQKLEEQMNKLRKIQNHIQTLKKWMAEVDVFLKEEWPALGDSEILKKQLKQCRLLVSDIQTIQPSLNSVNEEGQKIKNEAEPEFASRLETELKELNTQWDHMCQQVYARKEALKEELEKNEELKREIERLEEENRELERKIEYLKR | −12.5 |
| **Dy_3_BB*spyT*46** | Segments in order: **SpyTag**-P4SHb-Dy_3_-P6SHb-**HisTag**  M**AHIVMVDAYKPTK**GSGGSGGGSGKIEELKRKIEKLKRENERLERENEWLEREIIAFYNQLQQLEQMTTTAENWLKIQPTTPSEPTAIKSQLKICKDEVNRLSDLQPQIERLKIQSIALKEKGQGPMFLDADFVAFTNHFKQVFSDVQAREKELQTIFDTLPPMRYQETMSAIRTWVQQSETKLSIPQLSVTDYEIMEQRLEELQALQSSLQEQQSELYYLSTTVKEMSKKAPSEISRKYQSEFEEIEERWKKLSSQLVEHCQKLEEQMNKLRKIQNHIQTLKKWMAEVDVFLKEEWPALGDSEILKKQLKQCRLLVSDIQTIQPSLNSVNEEGQKIKNEAEPEFASRLETELKELNTQWDHMCQQVYARKEALKEELEKNEELKREIERLEEENRELERKIEYLKRGSG**HHHHHHHH** | −11.5 |
| **mNG*spyC*** | Segments in order: **HisTag**-mNG-**SpyCatcher**  M**HHHHHHHH**GSGVSKGEEDNMASLPATHELHIFGSINGVDFDMVGQGTGNPNDGYEELNLKSTKGDLQFSPWILVPHIGYGFHQYLPYPDGMSPFQAAMVDGSGYQVHRTMQFEDGASLTVNYRYTYEGSHIKGEAQVKGTGFPADGPVMTNSLTAADWSRSKKTYPNDKTIISTFKWSYTTGNGKRYRSTARTTYTFAKPMAANYLKNQPMYVFRKTELKHSKTELNFKEWQKAFTDVMGMDELYKGSGGSG**DSATHIKFSKRDEDGKELAGATMELRDSSGKTISTWISDGQVKDFYLYPGKYTFVETAAPDGYEVATAITFTVNEQGQVTVNG** | −5.1 |
| **mRFP*spyC*** | Segments in order: **HisTag**-**mRFP**-**SpyCatcher**  M**HHHHHHHH**GSG**ASSEDVIKEFMRFKVRMEGSVNGHEFEIEGEGEGRPYEGTQTAKLKVTKGGPLPFAWDILSPQFQYGSKAYVKHPADIPDYLKLSFPEGFKWERVMNFEDGGVVTVTQDSSLQDGEFIYKVKLRGTNFPSDGPVMQKKTMGWEASTERMYPEDGALKGEIKMRLKLKDGGHYDAEVKTTYMAKKPVQLPGAYKTDIKLDITSHNEDYTIVEQYERAEGRHSTGA**GSGGSGGSG**DSATHIKFSKRDEDGKELAGATMELRDSSGKTISTWISDGQVKDFYLYPGKYTFVETAAPDGYEVATAITFTVNEQGQVTVNG** | −10.2 |
| **HUHsfGFP_PAINT_** | Segments in order: **HUH**-**sfGFP**-**SpyCatcher**-**HisTag**  M**AKSGNYSYKRWVFTINNPTFEDYVHVLEFCTLDNCKFAIVGEEKGANGTPHLQGFLNLRSNARAAALEESLGGRAWLSRARGSDEDNEEYCAKESTYLRVGEPVSKGRSS**SG**KGEELFTGVVPILVELDGDVNGHKFSVRGEGEGDATIGKLTLKFICTTGKLPVPWPTLVTTLTYGVQCFSRYPDHMKRHDFFKSAMPEGYVQERTISFKDDGKYKTRAVVKFEGDTLVNRIELKGTDFKEDGNILGHKLEYNFNSHNVYITADKQKNGIKANFTVRHNVEDGSVQLADHYQQNTPIGDGPVLLPDNHYLSTQTVLSKDPNEKRDHMVLHEYVNAAGIT**GSGGSG**DSATHIKFSKRDEDGKELAGATMELRDSSGKTISTWISDGQVKDFYLYPGKYTFVETAAPDGYEVATAITFTVNEQGQVTVNG**GSG**HHHHHHHH** | −10.3 |
| **Dy_3_BB*Sw*55** | Segments in order: **HisTag**-P5SHb_3H3_-Dy_3_-P5SHb_3H3_  M**HHHHHHHH**GSGENRELEEKIRELKEKNEEHHREIKYLEEEIIAFYNQLQQLEQMTTTAENWLKIQPTTPSEPTAIKSQLKICKDEVNRLSDLQPQIERLKIQSIALKEKGQGPMFLDADFVAFTNHFKQVFSDVQAREKELQTIFDTLPPMRYQETMSAIRTWVQQSETKLSIPQLSVTDYEIMEQRLEELQALQSSLQEQQSELYYLSTTVKEMSKKAPSEISRKYQSEFEEIEERWKKLSSQLVEHCQKLEEQMNKLRKIQNHIQTLKKWMAEVDVFLKEEWPALGDSEILKKQLKQCRLLVSDIQTIQPSLNSVNEEGQKIKNEAEPEFASRLETELKELNTQWDHMCQQVYARKEALKEELEENRELEEKIRELKEKNEEHHREIKYLEE | −20.6 |
| **Dy_3_BB*Sw*66** | Segments in order: **HisTag**-P6SHb_3H3_-Dy_3_-P6SHb_3H3_  M**HHHHHHHH**GSGKNEELKREIERLEEENREHERKIEYLKREIIAFYNQLQQLEQMTTTAENWLKIQPTTPSEPTAIKSQLKICKDEVNRLSDLQPQIERLKIQSIALKEKGQGPMFLDADFVAFTNHFKQVFSDVQAREKELQTIFDTLPPMRYQETMSAIRTWVQQSETKLSIPQLSVTDYEIMEQRLEELQALQSSLQEQQSELYYLSTTVKEMSKKAPSEISRKYQSEFEEIEERWKKLSSQLVEHCQKLEEQMNKLRKIQNHIQTLKKWMAEVDVFLKEEWPALGDSEILKKQLKQCRLLVSDIQTIQPSLNSVNEEGQKIKNEAEPEFASRLETELKELNTQWDHMCQQVYARKEALKEELEKNEELKREIERLEEENREHERKIEYLKR | −14.7 |

**Table S3:** **SEC-MALS measurements of isolated protein constructs.**

| **Protein name** | **Theoretical value (kDa)** | **Measured value (kDa)** |
| --- | --- | --- |
| **Dy_3_-neg** | 39.6 | 40.0 (± 0.6 %) |
| **Dy_3_BB3** | 43.4 | 43.5 (± 0.6 %) |
| **Dy_3_BB4** | 43.5 | 42.1 (± 0.7 %) |
| **Dy_3_BB6** | 43.6 | 41.8 (± 0.5 %) |
| **Dy_3_BB5** | 43.4 | 49.7 (± 0.4 %) |
| **Dim-Dy_3_BB3:4** | 86.8 | 84.1 (± 0.5 %) |
| **Dim-Dy_3_BB5:6** | 86.8 | 74.6 (± 0.5 %) |
| **Dy_3_BB55** | 47.1 | 52.2 (± 0.7 %) |
| **Dy_3_BB66** | 47.2 | 56.6 (± 0.6 %) |
| **Dy_3_BB53** | 47.1 | 50.7 (± 0.7 %) |
| **Dy_3_BB46** | 47.2 | 50.5 (± 0.5 %) |
| **Dy_3_BB*mNG*53** | 74.1 | 82.5 (± 0.6 %) |
| **Dy_3_BB*mNG*46** | 74.2 | 83.0 (± 0.6 %) |
| **Dy_3_BB*Sw*55** | 47.2 | 47.7 (± 2.1 %) |
| **Dy_3_BB*Sw*66** | 47.3 | 50.7 (± 2.4 %) |

**Table S4:** **List of primers used for Gibson assembly (GA).**

| **Primer name** | **Nucleotide sequence** |
| --- | --- |
| **pET-41a(+)_for** | TAATGACCTAGGCTGCTAAACAAAGCC |
| **pET-41a(+)_rev** | ATGTATATCTCCTTCTTAAAC |

**Figure S1:** **Protein engineering and structural analysis of the spectrin repeat-based scaffolding unit.**

(**A**) The amino acid sequence of the Dy_3_ wild-type (R5-R7). Gly surface residues are bolded and highlighted in yellow. The molecular model of the Dy_3_ wild-type represents a tandem repeat of three spectrin domains, each composed of three α-helices. The expected end-to-end distance is ~15 nm and the diameter is ~2.5 nm. (**B**) The amino acid sequence of the Dy_3_-neg. Mutated Glu surface residues are bolded and highlighted in red. The molecular model of Dy_3_-neg displays the same structural features as the Dy_3_ wild-type. (**C**) Calculated net charge values at pH 7.4 for Dy_3_ wild-type and Dy_3_-neg.

**Figure S2:** **Biophysical characterization of the Dy_3_-neg.**

(**A**) SDS PAGE analysis of the purification process. Marked bands (black arrow) correspond to the target protein in whole cell lysate (lane 1), soluble fraction (lane 2), Ni-NTA eluate (lane 3), and SEC fractions after the purification. Protein markers are denoted with M. (**B**) SEC-MALS chromatogram with an indicated molecular weight of the Dy_3_-neg (black line), calculated from the light scattering data. (**C**) CD analysis of Dy_3_-neg. The CD was measured at 25 °C (black line), after thermal denaturation at 90 °C (red line), and after cooling the sample back to 25 °C (blue line). CD signal is shown as mean residue ellipticity (MRE). (**D**) The thermal denaturation curve was measured at 222 nm as a function of temperature (4 to 90 °C). Melting temperature (*T*_m_) was determined by fitting the experimental measurements with a thermodynamic sigmoidal curve in the Origin 2018 software. (**E**) DLS analysis of the Dy_3_-neg in the buffer solution.

**Figure S3:** **SAXS structural characterization of the Dy_3_-neg.**

(**A**) The experimental SAXS profile (black trace) of Dy_3_-neg and theoretical scattering (red trace) calculated for the model structure shown in panel (**C)**. (**B**) The pair-distance distribution function, P(r), was obtained by the SAXS experiments. (**C**) SAXS *ab initio* reconstruction superimposed on the molecular model of the Dy_3_-neg exhibiting the best fit to the experimental SAXS data (χ2 = 1.85). Scale bar = 5 nm.

**Figure S4: Structural features of the protein block design.**

(**A**) Visual depiction of the AF2 molecular model illustrating dimer formation (Dim-Dy_3_BB3:4), providing insights into the structural characteristics of the proposed protein block design. CC-forming peptide P3SHb is shown in light blue, the P4SHb is depicted in red and the Dy_3_-neg scaffold is presented in gray. This specific model was selected for clarity purposes. The same protein design principles were applied to all building blocks used in this study. (**B**) A detailed view of the joining section between the two interacting building blocks (Dy_3_BB3 and Dy_3_BB4). The molecular model displays the preserved α-helix continuity with a seamless transition from the terminal helix of the spectrin scaffolding domain to the CC-forming peptide and an appropriate frame to ensure the filamentous extension.

**Figure S5: Coiled-coil (CC) stabilization via electrostatic interactions.**

(**A-B**) Sequence and AF2 models of P3:P4SHb and P5:P6SHb CC motifs. Peptides P3SHb (light blue), P4SHb (light red), P5SHb (orange), and P6SHb (green) are displayed in their representative colors. Intrahelical stability is enhanced by introducing oppositely charged residues at the *b*, *c* and *f* positions (amino acid residues highlighted in red). Orthogonality of the pairing peptides is facilitated by oppositely charged residues at positions *e* and *g* (amino acid residues highlighted in yellow). Involved electrostatic interactions are depicted with green dashed lines.

**Figure S6: Purification analysis and biophysical characterization of Dy_3_BB3 and Dy_3_BB4.**

(**A**) SDS PAGE of purified monofunctional building blocks Dy_3_BB3 (lane 1) and Dy_3_BB4 (lane 2). The protein marker is denoted with M. (**B, D**) CD analysis of Dy_3_BB3 and Dy_3_BB4. The CD was measured at 25 °C (black line), after thermal denaturation at 90 °C (red line), and after cooling the sample back to 25 °C (blue line). CD signal is shown as mean residue ellipticity (MRE). (**C, E**) Thermal denaturation curves for Dy_3_BB3 and Dy_3_BB4 were measured at 222 nm as a function of temperature (4 to 90 °C).

**Figure S7: Purification analysis and biophysical characterization of Dy_3_BB6 and Dy_3_BB5.**

(**A**) SDS PAGE of purified monofunctional building blocks Dy_3_BB6 (lane 1) and Dy_3_BB5 (lane 2). The protein marker is denoted with M. (**B, D**) CD analysis of Dy_3_BB6 and Dy_3_BB5. The CD was measured at 25 °C (black line), after thermal denaturation at 90 °C (red line), and after cooling the sample back to 25 °C (blue line). CD signal is shown as mean residue ellipticity (MRE). (**C, E**) Thermal denaturation curves for Dy_3_BB6 and Dy_3_BB5 were measured at 222 nm as a function of temperature (4 to 90 °C).

**Figure S8: DLS and AFM characterization of CC-facilitated dimer assembly.**

(**A**) Schematic representation of extended dimers Dim-Dy_3_BB3:4 and Dim-Dy_3_BB5:6. The end-to-end distance is expected to be ~35 nm. (**B-C**) DLS analysis confirmed the presence of larger particles corresponding to Dim-Dy_3_BB3:4 and Dy_3_BB5:6 (red line). The black line represents Dy_3_BB3 (**B**) and Dy_3_BB5 (**C**). Meanwhile, the blue line represents Dy_3_BB4 (**B**) and Dy_3_BB6 (**C**). (**D-E**) AFM micrographs of Dim-Dy_3_BB3:4 (**D**) and Dim-Dy_3_BB5:6 (**E**). Slightly bent assemblies of expected end-to-end distances were detected. Representable structures are highlighted with white dashed circles.

**Figure S9: Schematic explanation of bifunctional building block design and proposed mechanism of head-to-tail polymerization.**

(**A**) Schematic representation of bifunctional protein blocks Dy_3_BB55 and Dy_3_BB66 with identical CC-forming peptide arrangement. Due to the steric hindrance caused by the bulkiness of the central Dy_3_-neg scaffolding domain, AF2 cannot predict intermolecular dimerization along the lateral axis. However, head-to-tail polymerization in the filamentous conformation along the longitudinal axis is favored. The CC linking motif P5:P6SHb is highlighted with a black dashed rectangle. (**B**) Schematic representation of bifunctional building blocks Dy_3_BB53 and Dy_3_BB46 with alternate peptide arrangement. An equimolar mix of both subunits led to the predicted head-to-tail polymerization, confirmed by AF2 molecular modeling. This arrangement preserved free reactive ends (sticky ends) during head-to-tail polymer assembly. CC linking motifs P3:P4SHb and P5:P6SHb are marked with black dashed rectangles.

**Figure S10: Purification analysis and biophysical characterization of Dy_3_BB55 and Dy_3_BB66.**

(**A**) SDS PAGE of purified bifunctional building blocks Dy_3_BB55 (lane 1) and Dy_3_BB66 (lane 2). The protein marker is denoted with M. (**B, D**) CD analysis of Dy_3_BB55 and Dy_3_BB66. The CD was measured at 25 °C (black line), after thermal denaturation at 90 °C (red line), and after cooling the sample back to 25 °C (blue line). CD signal is shown as mean residue ellipticity (MRE). (**C, E**) Thermal denaturation curves for Dy_3_BB55 and Dy_3_BB66 were measured at 222 nm as a function of temperature (4 to 90 °C).

**Figure S11: Purification analysis and biophysical characterization of Dy_3_BB53 and Dy_3_BB46.**

(**A**) SDS PAGE of purified bifunctional building blocks Dy_3_BB53 (lane 1) and Dy_3_BB46 (lane 2). The protein marker is denoted with M. (**B, D**) CD analysis of Dy_3_BB53 and Dy_3_BB46. The CD was measured at 25 °C (black line), after thermal denaturation at 90 °C (red line), and after cooling the sample back to 25 °C (blue line). CD signal is shown as mean residue ellipticity (MRE). (**C, E**) Thermal denaturation curves for Dy_3_BB53 and Dy_3_BB46 were measured at 222 nm as a function of temperature (4 to 90 °C).

**Figure S12: AFM imaging of individual proteins.**

AFM micrographs of the central spectrin repeat-based scaffolding unit Dy_3_-neg and bifunctional building blocks (Dy_3_BB55, Dy_3_BB66, Dy_3_BB53, Dy_3_BB46, and Dy_3_BB*spyT*46), utilized for the construction of designed filamentous protein polymers. All images were visualized using a dry-tapping mode experiment under the same magnification.

**Figure S13: AFM imaging of Poly-Dy_3_BB55:66.**

(**A**) AFM micrograph of self-assembled polymers under low magnification. (**B**) Magnified images display numerous polymer assemblies in circular (white dashed circles) or extended (white dashed squares) conformations. All images were visualized using a dry-tapping mode experiment.

**Figure S14: Image quantification of Poly-Dy_3_BB55:66.**

The first column shows the original AFM images, the second column presents the merged segmented images highlighting aggregated (red) and organized filamentous and circular structures (blue), and the third column displays skeletonized images of all objects. Quantified values (area %) are presented in the bottom right corner of individual AFM images (black rectangle). Marking S corresponds to organized structures, meanwhile, A corresponds to aggregates.

**Figure S15: Particle analysis of Poly-Dy_3_BB55:66.**

(**A**) Histogram representing the distribution of total length-weighted lengths, derived from non-segmented (total) skeletonized AFM images in **Figure S14**. (**B**) Histogram representing the distribution of length-weighted lengths per population, derived from segmented AFM images (blue objects only) in **Figure S14**. In (**A**) and (**B**) length-weighted counts denote the sum of all filament’s lengths in each bin. (**C**) Height analysis distribution of organized structures (per population). (**D**) Calculated average area (%) for aggregates (A) and organized structures (S) acquired from the segmented images in **Figure S14**. (**E**). Calculated length-weighted average lengths (total and per population) and height values for the analyzed images in **Figure S14**.

**Figure S16: Morphology analysis of Poly-Dy_3_BB55:66.**

(**A**) AFM micrographs of self-assembled polymers under low and high magnifications. (**B**) Height analysis (distance analysis) measured along the white dashed line in (**A**) (detailed view).

**Figure S17: Negative stain TEM imaging of Poly-Dy_3_BB55:66.**

ns-TEM images of self-assembled protein polymers under different magnifications. White arrows indicate rigid rods composed of bundled single-chain filaments, while yellow arrows depict the polymer thickness at the selected section.

**Figure S18: AFM imaging of Poly-Dy_3_BB53:46.**

AFM micrographs of protein polymers under various magnifications. To demonstrate the efficiency and robustness of polymer self-assembly, numerous fields of view were acquired. The micrographs display several μm-long rigid rods and numerous entangled structures. All images were visualized using a dry-tapping mode experiment.

The figure continues on the next page.

**Figure S19: Image quantification of Poly-Dy_3_BB53:46.**

The first column shows the original AFM images, the second column presents the merged segmented images highlighting aggregated (red) and filamentous structures (blue), and the third column displays skeletonized images of all objects. Quantified values (area %) are presented in the bottom right corner of individual AFM images (black rectangle). Marking F corresponds to filamentous structures, meanwhile, A corresponds to aggregates.

**Figure S20: Particle analysis of Poly-Dy_3_BB53:46**

(**A**) Histogram representing the distribution of total length-weighted lengths, derived from non-segmented (total) skeletonized AFM images in **Figure S19**. (**B**) Histogram representing the distribution of length-weighted lengths per population, derived from segmented AFM images (blue objects only) in **Figure S19**. In (**A**) and (**B**) length-weighted counts denote the sum of all filament’s lengths in each bin. (**C**) Height analysis distribution of selected filamentous objects (per population). (**D**) Calculated average area (%) for aggregates (A) and filaments (F) acquired from the segmented images in **Figure S19**. (**E**). Calculated length-weighted average lengths (total and per population) and height values for the analyzed images in **Figure S19**.

**Figure S21: Morphology analysis of Poly-Dy_3_BB53:46.**

(**A**) AFM micrographs of self-assembled polymers under low and high magnifications. Well-defined spectrin repeat-based rigid rods are magnified in the detailed (magnified) view. All images were visualized using a dry-tapping mode experiment. (**B**) Height analysis (distance analysis) measured along the white dashed line in (**A**).

**Figure S22: Negative stain TEM imaging of Poly-Dy_3_BB53:46.**

ns-TEM images of self-assembled protein polymers under different magnifications. White arrows indicate rigid rods (bundles of single-chain filaments), red arrows indicate individual single chains and yellow arrows display the polymer thickness at three different sections.

**Figure S23: Self-assembly of functionalized rods Poly-Dy_3_BBmNG53:46.**

(**A**) Schematic representation of the self-assembly into functionalized single-chain filaments and spatial positioning of functional domains (mNeonGreen). End-to-end distances are indicated by black arrows. The direct fusion of mNeonGreen via a flexible linker does not impair the CC formation (detailed view). (**B**) The AF2 molecular model of the Poly-Dy_3_BB*mNG*53:46. (**C**) Photograph of the protein mixture after polymerization.

**Figure S24: Purification analysis and biophysical characterization of Dy_3_BBmNG53 and Dy_3_BBmNG46.**

**(A**) SDS PAGE of purified bifunctional building blocks Dy_3_BB*mNG*53 (lane 1) and Dy_3_BB*mNG*46 (lane 2). The protein marker is denoted with M. (**B, D**) CD analysis of Dy_3_BB*mNG*53 and Dy_3_BB*mNG*46. The CD was measured at 25 °C (black line), after thermal denaturation at 90 °C (red line), and after cooling the sample back to 25 °C (blue line). CD signal is shown as mean residue ellipticity (MRE). (**C, E**) Thermal denaturation curves for Dy_3_BB*mNG*53 and Dy_3_BB*mNG*46 were measured at 222 nm as a function of temperature (4 to 90 °C). (**F**) DLS analysis of Dy_3_BB*mNG*53 (red line) and Dy_3_BB*mNG*46 (green line). (**G**) SEC-MALS chromatogram with indicated molecular weights of individual protein blocks Dy_3_BB*mNG53* (red line) and Dy_3_BB*mNG*46 (green line), calculated from the light scattering data.

**Figure S25:** **Self-assembly of functionalized rods Poly-Dy_3_BBmNG46:53.**

(**A**) The AF2 molecular model of Poly-Dy3BB*mNG*46:53. (**B**) Native PAGE analysis of high-molecular-weight protein polymers, which were too large to be well-separated on the gel (lane 3, black arrow). Lanes 1 and 2 represent the mobility patterns of Dy_3_BB53 and Dy_3_BB*mNG*46. (**C**) Photograph of the protein mixture after polymerization.

**Figure S26: Negative stain TEM images of Poly-Dy_3_BBmNG53:46.**

ns-TEM images of functionalized protein polymers under different magnifications. White arrows indicate bundled spectrin repeat-based rods. Numerous short and flexible single chains can be observed in the background of the images.

**Figure S27: TIRFM images of Poly-Dy_3_BBmNG53:46.**

The images display mNeonGreen-decorated protein polymers, exhibiting a strong fluorescence signal along the rods' longitudinal axes.

**Figure S28:** **Indirect functionalization and TIRFM imaging of Poly-Dy_3_BBspyT46:53 rods.**

(**A**) Schematic representation of the head-to-tail polymerization into single-chain filaments (Dy_3_BB53 and Dy_3_BBspyT46) with exposed short SpyTag peptides at N-terminal ends (Dy_3_BBspyT46) and the spatially-defined ligation of mNeonGreen domains via the SpyCatcher-SpyTag system. (**B**) TIRFM images display long, well-defined protein polymers with strong fluorescence signals along the rods’ longitudinal axes.

**Figure S29: Co-immobilization of two fluorescence proteins on Poly-Dy_3_BBspyT46:53 rods.**

(**A**) Schematic representation of the spatially-controlled ligation of mNG and mRFP fusion proteins (equimolar ratio) on a single-chain filament using the SpyCatcher-SpyTag system. (**B**) TIRFM images of polymers in three different fields of view, displaying green, red and overlayed fluorescence signals along their longitudinal axes.

**Figure S30:** **Super-resolution imaging with DNA-PAINT.**

(**A**) Schematic representation of the DNA-PAINT experiment. Poly-Dy_3_BB*spyT*46:53 rods were functionalized with HUHsfGFP_PAINT_ construct via a SpyCatcher-SpyTag ligation system. N-terminal single-stranded DNA-binding domain (HUH) in fusion with superfolder GFP (sfGFP) and SpyCatcher was conjugated with a docking DNA strand (docker; gray color). The domain periodicity was detected via transient interactions with a complementary DNA strand (imager; red color), coupled to the atto655 fluorescence label. The separation can range ± 10 nm due to the flexible attachment of the decorated protein domains. (**B**) A complete field of view under TIRFM. The analyzed rod in **Figure 5F** is marked with a white dashed rectangle.

**Figure S31: Super-resolution imaging of co-immobilized rods with DNA-PAINT.**

(**A**) Schematic representation of the DNA-PAINT experiment. Poly-Dy_3_BB*spyT*46:53 rods were functionalized with the HUHsfGFP_PAINT_ construct and mRFP*spyC* in the equimolar ratio. The protein periodicity was detected via transient interactions with a complementary DNA strand (imager; red color), coupled to the atto655 fluorescence label. (**B**) TIRFM images of the selected field of view, displaying green, red and overlayed fluorescence signals along the rods' longitudinal axes. The analyzed rod section is marked with a white dashed rectangle (the same position on all images). (**C**) DNA-PAINT super-resolution imaging of Poly-Dy_3_BB*spyT*46:53 rods. Individual attachment sites with expected spacing between labeled proteins can be resolved (black arrows). The measured distances between displayed proteins (bright dots) are shown next to the corresponding arrows. The expected theoretical distance between presented proteins is noted in the upper right corner of the image. The separation can range ± 10 nm due to the flexible attachment of the decorated protein domains.

**Figure S32: Purification analysis and biochemical characterization of Dy_3_BBSw55 and Dy_3_BBSw66.**

(**A**) SDS PAGE of purified monofunctional building blocks Dy_3_BB*Sw*55 (lane 1) and Dy_3_BB*Sw*66 (lane 2). The protein marker is denoted with M. (**B, D**) CD analysis of Dy_3_BB*Sw*55 and Dy_3_BB*Sw*66. The CD was measured at 25 °C (black line), after thermal denaturation at 90 °C (red line), and after cooling the sample back to 25 °C (blue line). CD signal is shown as mean residue ellipticity (MRE). (**C, E**) Thermal denaturation curves for Dy_3_BB*Sw*55 and Dy_3_BB*Sw*66 were measured at 222 nm as a function of temperature (4 to 90 °C).

**Figure S33: Morphology analysis of Poly-Dy_3_BBSw55:66.**

(**A**) AFM micrograph analysis of self-assembled Zn(II)-responsive protein polymers. The image was visualized using a dry-tapping mode experiment. (**B**) Height and length analysis (distance analysis) measured along the white dashed line in (**A**).

**Figure S34: Image quantification of Poly-Dy_3_BBSw55:66.**

The first column shows the original AFM image, the second column presents the merged segmented image highlighting aggregated (red) and defined intertwined filamentous structures (blue), and the third column displays the skeletonized image of selected objects. Quantified values (area %) are presented in the bottom right corner of the AFM image (black rectangle) and the table below. Marking S corresponds to organized structures, meanwhile, A corresponds to aggregates.
